# Whole genome sequencing of *Castanea mollissima* and molecular mechanisms of sugar and starch synthesis

**DOI:** 10.3389/fpls.2024.1455885

**Published:** 2024-10-25

**Authors:** Rongchen Li, Xiaolu Huang, Liping Yang, Jianming Liao, Xiaojuan Wei, Junji Li, Guangyu Zeng, Dan Liu, Zhuogong Shi, Zhiheng Zhao

**Affiliations:** ^1^ Guangxi Forestry Research Institute, Guangxi Forestry Research Institute, Guangxi Forestry Laboratory, Guangxi Key Laboratory of Special Non-wood Forests Cultivation and Utilization, Nanning, China; ^2^ College of Biological Sciences and Biotechnology, National Engineering Research Center for Forest Tree Breeding and Ecological Remediation, Key Laboratory of Genetics and Breeding in Forest Trees and Ornamental Plants, Ministry of Education, The Tree and Ornamental Plant Breeding and Biotechnology Laboratory of National Forestry and Grassland Administration, Beijing Forestry University, Beijing, China; ^3^ Shandong Provincial Center of Forest and Grass Germplasm Resources, Jinan, China; ^4^ Research Center for Plateau Characteristic Agriculture in Northeast Yunnan, College of Agriculture and Life Sciences, Zhaotong University, Zhaotong, China

**Keywords:** chestnut, telomere-to-telomere genome assembly, time-ordered gene co-expression network, pollen xenia, sugar and starch metabolism

## Abstract

The chestnut tree exhibits self-incompatibility, where the selection of the male parent (pollen xenia) significantly affects seed starch metabolism, as well as fruit yield and quality. Despite its importance, the molecular mechanisms underlying pollen xenia remains largely unknown. In this study, we utilized the ‘Lan You’ variety of *C. mollissima* to construct a high-quality reference genome. As a result, a first Telomere-to-telomere (T2T) gap-free genome for this species was successfully assembled. A total of 560 transcription factors and 22 structural genes were identified as consistent across the TO-GCNs, indicating a consistent regulation pattern in the co-expression of genes involved in starch accumulation. These networks were further divided into three sub-networks: T1, T2, and T3. Among these, the T1 and T2 sub-networks exhibited a higher number of structural genes with consistent regulation patterns and were closely associated with sugar biosynthesis. The gene SBE (*Camol08G0254600*) was identified as the hub gene with the highest degree of connectivity, encoding a key rate-limiting enzyme in the amylopectin biosynthesis pathway. This study provides a foundation for further research on *C. mollissima* population genetics, genetic improvement, and strategies aimed at enhancing yield and quality.

## Introduction

*Castanea mollissima*, a species within the genus *Castanea* and the family Fagaceae, is a vital economic tree species with approximately 300 varieties native to China and the Korean peninsula. It is primarily found in the temperate and subtropical zones of the Northern Hemisphere (Sun et al., 2020). Renowned as the “king of dry fruits”, it is celebrated for its distinctive flavor, exceptional nutritional content, and significant economic and medicinal value (Xing et al., 2019). Starch, the primary metabolite in *C. mollissima*, consists of amylose and amylopectin, contributing to 47%-80% of the nut’s dry weight (Shi et al., 2021), and significantly influences its taste. In China, *C. mollissima* exhibits low fruit set rates, suboptimal quality, low sugar content, and insufficient glutinousness, highlighting an urgent need for improvement in both yield and quality (Li et al., 2021). Enhancing the starch quality in *C. mollissima* through the pollen xenia effect to select high-quality varieties and alter the types and contents of starch is of utmost importance. Current research of *C. mollissima* starch primarily focuses on physiological and biochemical aspects, identifying several genes associated with starch synthesis. However, investigations into the molecular regulatory mechanisms of starch and sugar metabolic pathways during *C. mollissima* growth have lagged behind. Genome sequencing has the potential to significantly broaden the scope and depth of research, providing powerful tools for the study of important plant traits, such as fruit quality (Wang et al., 2020; Meadows and Lindblad-Toh, 2017; Paterson et al., 2009; Ma et al., 2021). Additionally, it facilitates the exploration of abundant genetic resources in plants, aids in understanding their origin and evolution, genetics, and improvement, and promotes the identification of candidate genes associated with key traits and the development of molecular markers (Chen et al., 2020; Derelle et al., 2006; Fan et al., 2020; Goff et al., 2002).

Recent advancements in sequencing technology have facilitated a more comprehensive understanding of whole-genome information across numerous plant species (Chang et al., 2019; Zhang et al., 2021; Goodwin et al., 2016). Long-reads sequencing technology is utilized for telomere-to-telomere (T2T) genome assembly (Li and Durbin, 2024). In recent years, the genomes of seven *Castanea mollissima* varieties have been sequenced (Xing et al., 2019; Sun et al., 2020; Wang et al., 2020; Staton et al., 2020; Hu et al., 2022). However, high-quality T2T genomes of *C. mollissima* are still lacking. The pollen xenia effect has a direct impact on fruit quality and seed characteristics. Chestnuts also exhibit a significant pollen xenia effect. Research has shown a significant correlation between the xenia effect in chestnuts and their starch content, including the amylopectin content. Additionally, it has been observed that the content of soluble starch is significantly and positively correlated with the activity of soluble starch synthase (SSS) and granule-bound starch synthase (GBSS) during the growth and development of chestnut kernels (Li et al., 2021).

By comparing the transcriptome of chestnut kernels at different developmental stages, no differentially expressed genes were identified related to cytoplasmic AGP were found, suggesting that the starch biosynthesis pathway in chestnut is similar to that in potato tubers and *Arabidopsis* leaves, but differs from the genetic pathway observed in maize endosperm (Zhang et al., 2015). In addition, our study identified and characterized the activities of two starch branching enzymes, CmSBEI and CmSBEII. We observed that the expression activities of both CmSBEI and CmSBEII peaked at 74 DAP (Day After Pollination). Correspondingly, the gene expression levels of these two CmSBE isoforms exhibited similar trends, with an increase starting from 64 DAP and reaching their highest levels at 77 DAP. This indicates that CmSBE enzymes are involved in the synthesis of amylopectin and influence its content in the kernel (Chen et al., 2017). The research on the molecular regulatory mechanism of chestnut pollen xenia is currently limited. However, high-throughput transcriptome sequencing combined with co-expression network analysis offers promising approaches for constructing a comprehensive regulatory network of chestnut pollen xenia.

In this study, the local variety of *C. mollissima*, ‘Lan You’, renowned for its significant economic traits, was selected for whole-genome sequencing using Illumina and PacBio technologies. The complete assembly at the chromosomal level was achieved through the complementary use of Hi-C-assisted genome technology. Furthermore, comprehensive gene annotation and the identification of duplicated sequences were conducted. This comprehensive approach achieved telomere-to-telomere assemblies of *C. mollissima* genome, significantly enhancing the understanding of the species’ phylogenetic placement within angiosperms. This understanding provides crucial references for subsequent functional studies and the application of modern biotechnology for genetic modification. Consequently, it facilitates the elucidation of the genetic basis underlying the significant economic traits of *C. mollissima* and lays a solid foundation for molecular breeding and genotype selection in *C. mollissima*.

## Materials and methods

### Plant materials

**Figure 1:**
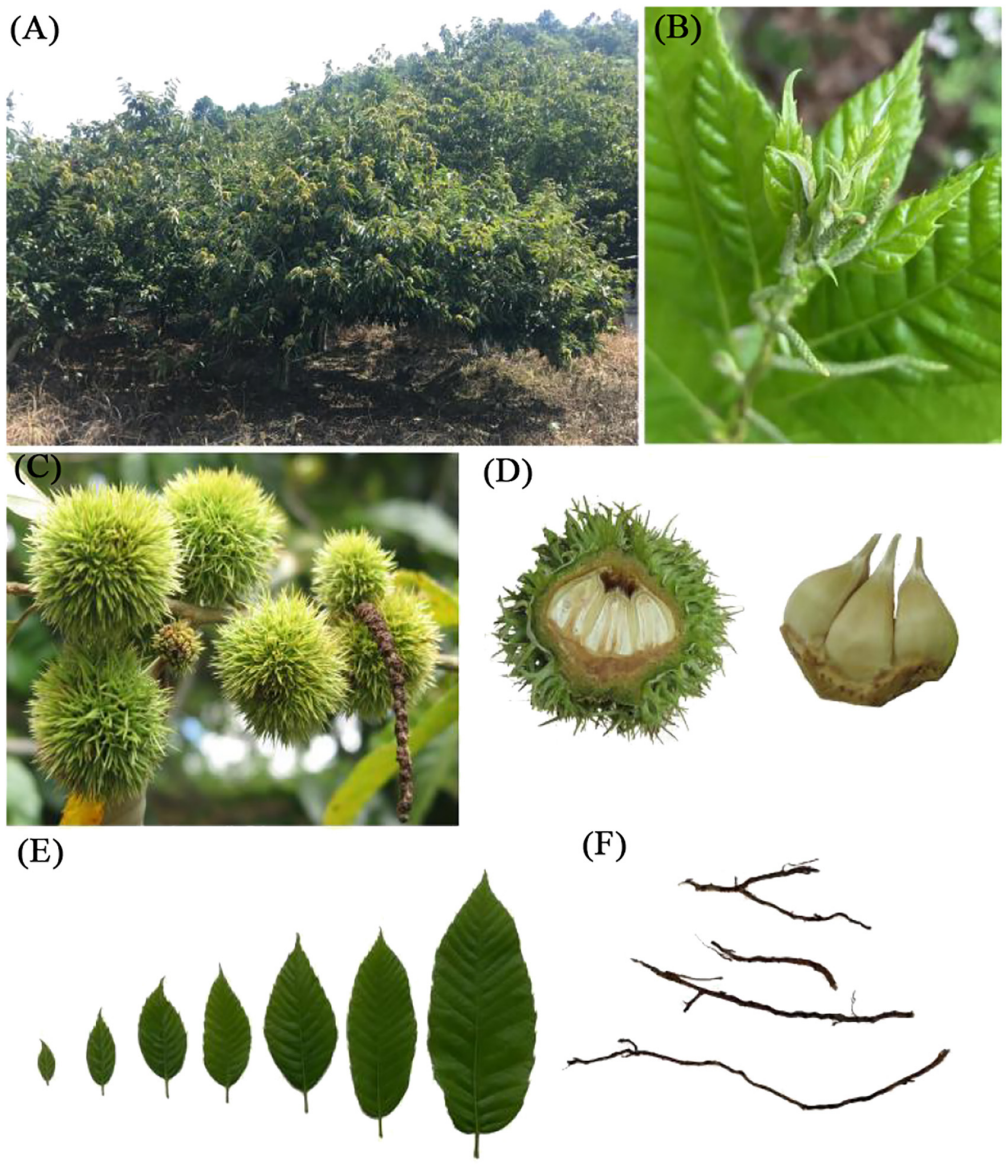
Experimental materials (Roots, stems, leaves and young fruits of chestnut). **(A)** chestnut tree; **(B)** stem; **(C, D)** young fruit; **(E)** leaves; **(F)** roots.

The experimental site was situated within the experimental forest farm of the Guangxi Forestry Research Institute. The study focused on a 5-year-old variety of *Castanea mollissima* known as ‘Lan You’ (LY), the primary cultivated chestnut variety in Guangxi. Various plant tissues, including fresh leaves, flower buds, roots, stems, and young fruits were carefully selected from the chestnut trees (**Figure 1**). Subsequently, these samples were rapidly frozen in liquid nitrogen and stored at -80°C to preserve their integrity.

### Whole genome sequencing

In this study, the entire genome of the chestnut was sequenced using a combination of PacBio HiFi, Hi-C, and Illumina sequencing platforms (Sangeret et al., 1977). High-quality genomic DNA was extracted from leaves using the CTAB method. Following rigorous quality control, the library was constructed and sequenced. For PacBio sequencing, a long-read SMRTbell library was constructed. After fragment screening and quantification, the library template was prepared along with the sequencing enzyme complex, and sequencing was performed on the PacBio Sequel II system. The Hifi reads were generated by the CCS program (https://github.com/PacificBiosciences/ccs). For Illumina sequencing, the genomic DNA was fragmented into 200 to 400 bp segments using ultrasonic shearing. The fragmented DNA ends were then blunted, and adapters were ligated to both ends. Following adapter ligation, the library fragments were screened through two rounds of magnetic bead capture, and a paired-end (2 × 150 bp) sequencing library was constructed. The entire genome was subsequently sequenced on the Illumina HiSeq platform.

### Genome assembly

The genome assembly in this study was accomplished through three sequential steps: primary assembly, Hi-C scaffolding, and subsequent optimization. Initially, PacBio HiFi long reads were assembled into contigs using hifiasm v0.15.1-r334 (Cheng et al., 2021) to produce the primary assembly for further analysis. In the second step, these contigs were anchored to 12 chromosomes using Hi-C data. The Hi-C data were first aligned to the preliminary genome assembly using Juicer (Durand et al., 2016), followed by chromosome assembly through the 3D-DNA scaffolding pipeline (Dudchenko et al., 2017). Manual adjustments were then performed with Juicebox (Durand et al., 2016), with a primary focus on refining chromosome boundary segmentation. Subsequently, the 3D-DNA pipeline was employed to re-scaffold the chromosomes, with further manual adjustments using Juicebox to correct boundaries, remove erroneous insertions, and adjust orientations. This process ultimately generated the chromosome frameworks and separate sequences, with gaps standardized to 100 bp in length. The third step focused on optimizing the genome assembly, which mainly involved gap filling and polishing. This optimization process included the following steps: (1) filling gaps using tgs-gapcloser (Xu et al., 2020) with HiFi long reads (parameters: –ne –min_match 1000); (2) assembling chloroplast and mitochondrial genomes using GetOrganelle (Jin et al., 2020); (3) performing three rounds of polishing with nextpolish (Hu et al., 2020) on second-generation short-read data; and (4) identifying redundancies by aligning scattered contig sequences with chromosome and organelle genome sequences using Redundans (Pryszcz and Gabaldon, 2016).

### Genome assembly completeness assessment

Four methods were employed in this study to evaluate the completeness of the genome assembly. First, the total genome size was compared with previously published values. Second, the contig N50 reached 47.97 Mb, indicating good continuity. Third, BUSCO (Benchmarking Universal Single-Copy Orthologs) (Simao et al., 2015) was used to assess the presence of 1,440 orthologous single-copy genes in embryophytes (Embryophyta). Finally, BWA (Li, 2013), Minimap2 (Li, 2018), and HISAT2 were utilized to align Illumina short reads, PacBio long reads, and RNA-Seq data to the genome, with read alignment rates and percentages of genome coverage computed (Ye et al., 2017; Zhao et al., 2023).

### Transcriptome sequencing and assembly

Total RNA was extracted using the QIAGEN RNeasy Plant Mini Kit (QIAGEN, USA). The integrity and concentration of the RNA were assessed using agarose gel electrophoresis and Nanodrop spectrophotometer. Subsequently, cDNA was synthesized via reverse transcription using the Transcript One-Step gDNA Removal and cDNA Synthesis SuperMix cDNA kit (TransGen Biotech, San Francisco, CA). RNA-Seq libraries were then generated using the mRNA-Seq Sample Preparation Kit (Illumina Inc., San Diego, CA, USA) and sequenced with paired-end reads on the Illumina HiSeq X Ten platform.

To construct a comprehensive chestnut transcriptome, this study employed three assembly strategies to analyze RNA-seq data from 18 sequenced samples of various tissues, including roots, stems, leaves, young fruits, buds, and flower buds. The strategies included: (1) *de novo* assembly using Trinity (Grabherr et al., 2011); (2) reference-guided assembly, where HISAT2 (Kim et al., 2015) was used to align RNA-seq data to the chestnut genome, followed by assembly using the genome-guided mode in Trinity and StringTie (Pertea et al., 2015); and (3) redundancy removal and final assembly. All transcript sequences were merged, and CD-HIT (Fu et al., 2012) was employed to remove redundancy based on 95% similarity and 95% coverage. Finally, the combined data were assembled into a set of transcript sequences using the PASA pipeline (Haas et al., 2003), which was used as the final transcriptome.

### Gene annotation

The *de novo* prediction of gene structure began with the PASA pipeline for initial annotations. Full-length genes were identified by comparison with reference proteins, and AUGUSTUS (Stanke et al., 2008) was then used to train the full-length gene set, followed by five rounds of optimization. The MAKER annotation process (Cantarel et al., 2008) was employed to predict gene structure as follows: (1) Repeat regions were masked using RepeatMasker; (2) AUGUSTUS performed *de novo* predictions on the repeat-masked genome; (3) BLASTN and TBLASTX were used to align the transcriptome sequence with the genome, and BLASTX aligned the protein sequence with the genome; (4) Exonerate v2.4.0 (Slater and Birney, 2005) optimized these alignments; (5) AUGUSTUS integrated the predicted gene model based on this evidence; and (6) UTR annotations were incorporated using transcriptome data. To enhance the accuracy of gene sequence prediction, EVidenceModeler (EVM) (Haas et al., 2008; Lowe and Eddy, 1997) was used to integrate MAKER and PASA annotations, generating consistent gene annotations. PASA subsequently upgraded the EVM annotations by adding UTR annotations and identifying alternative splicing events over two iterative rounds. Gene annotations with abnormal coding frames, such as those containing stop codons or ambiguous bases, and those lacking start or stop codons), as well as those less than 50 amino acids in length, were removed. This filtering step ensured high-quality gene structure annotations.

### Comparative genome and phylogenetic analysis

Gene family analysis was conducted using protein-coding genes from chestnut and ten other plant species, including two monocot species: rice (*Oryza sativa*) and maize (*Zea mays*); five Fagales species: chestnut (*Castanea mollissima*), oak (*Quercus robur*), white oak (*Quercus lobata*), cork oak (*Quercus suber*), and walnut (*Juglans regia*); and three additional species: potato (S*olanum tuberosum*), Arabidopsis (*Arabidopsis thaliana*), poplar (*Populus trichocarpa*), and soybean (*Glycine max*). For genes with multiple alternatively spliced transcripts, only the transcript with the longest coding region was retained for analysis (Van et al., 2018). Additionally, genes with protein sequences shorter than 50 amino acids were excluded. The filtered protein sequences were then aligned using BLASTP v2.2.26 with an E-value threshold of 1E-5, a similarity threshold of 30%, and a coverage threshold (alignment length divided by sequence length) of 30%. Based on the alignment results, the genes from the eleven species were clustered using the Markov cluster algorithm (MCL) in OrthoMCL v2.0.9 (Li et al., 2003). A total of 324 1:1:1 single-copy gene families (i.e., each species in the family has only one gene) were identified. The protein sequences from these families were fully aligned, and the single-copy genes were concatenated into a supergene. Codons from positions 1, 2, 3, 1 + 2, and fourfold degenerate (4d) sites were extracted for further analysis. Rice and maize were chosen as outgroups for constructing the evolutionary trees.

Utilizing the evolutionary tree results and previous research on evolutionary timelines, MCMCTree was employed to infer evolutionary times (Yang, 2007; Kim et al., 2014). Time calibration points were sourced from Timetree (http://www.timetree.org/), with three calibration points used: maize vs. rice (42 - 52 MYA), rice vs. Arabidopsis (115 - 308 MYA), and soybean vs. Arabidopsis (98 - 117 MYA). CAFE v4.2 (Han et al., 2013) was then used with default parameters to analyze the expansion and contraction of gene families. Gene families with large deviations were filtered out. Finally, GO enrichment analysis for the expanded gene families was performed using clusterProfiler v3.8.1 (Yu et al., 2012).

### Gene duplication

Gene family analysis was conducted using the protein-coding genes obtained from the genomes of chestnut and ten other plant species, which included two monocotyledonous species (Oryza sativa and Zea mays) five Fagales species (*Castanea mollissima*, *Quercus robur*, *Quercus lobata*, *Quercus suber*, and *Juglans regia*), as well as *Solanum tuberosum*, *Arabidopsis thaliana*, *Populus trichocarpa*, and *Glycine max*. Synteny analysis was conducted using gene pairs. Preliminary alignment was performed with BLASTP v2.2.26, followed by synteny identification using MCScanX (Wang et al., 2012). To identify synteny blocks between the chestnut and *Quercus robur* genomes, as well as between the chestnut and grape genomes, each synteny block was required to contain at least five genes. Based on the results of the homology alignment and collinearity analysis, *K*s values were calculated using the yn00 program, and WGDI (Sun et al., 2022) was employed for filtering, correction, and visualization of these Ks values.

### Pathway and genes associated with starch biosynthesis

To uncover the metabolic pathways and key genes associated with chestnut starch synthesis, protein sequence alignment was conducted using the Ensemble Enzyme Prediction Pipeline (E2P2) package (version 3.1) (https://gitlab.com/rhee-lab/E2P2). A comprehensive metabolic pathway database was established based on protein annotations obtained from the Plant Metabolic Network (https://www.plantcyc.org).

### RNA-seq analysis associated with pollen xenia

Previous studies have shown that Yongfeng 1♀ × Yongren Zao♂ (YFR) has high sugar and starch content, while Yongfeng 1♀ × Yimen 1♂ (YFM) has low sugar and starch content. To investigate the dynamic changes in sugar and starch content over time, three time points (T1, T2, and T3) were selected approximately 20, 10, and 0 days before chestnut maturity for both YFR and YFM. Differential expression analysis was performed across different time points using the original expression matrix. Utilizing DESeq2 in R (Love et al., 2014), three biological replicates from each time point were collectively analyzed, and all subgroups were compared to identify differentially expressed genes. The thresholds for significant differential expression were set at p.adjust < 0.05 and |log2FoldChange| > 1.

### Time-ordered gene co-expression network analysis

Genes exhibiting significant differential expression between any two groups were retained, resulting in a total of 11,622 significantly differentially expressed genes. Subsequently, the mean gene expression TPM (Transcripts Per Million) values for each time point group were calculated, and genes with a mean value greater than 0.5 in any group were selected. A total of 11,427 genes were ultimately chosen for constructing the temporal gene co-expression network. Furthermore, six content indicators were treated as genes, and their data were incorporated into the TPM expression matrix. The combined matrix served as the input file for constructing the temporal gene co-expression network using TOGCN (Langfelder and Horvath, 2008).

## Results

### Genome sequencing and assembly

**Figure 2:**
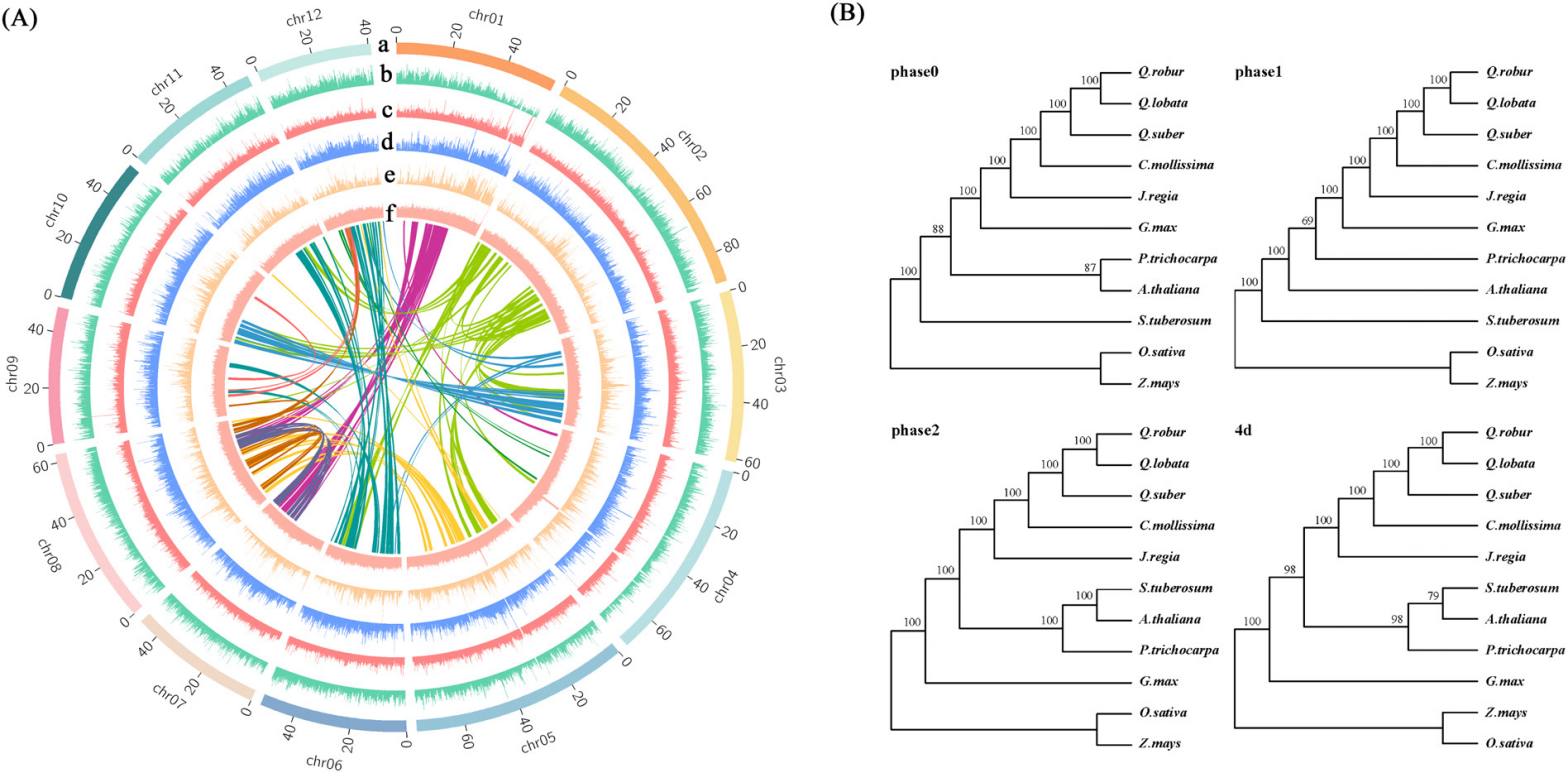
Genome sequencing analysis. **(A)** Collinearity and distributions of genome characteristics; a: Chromosome; b: Coding gene; c: TE/DNA; d: TE/Gypsy; e: TE/Copia; f: GC contents. **(B)** Phylogenetic trees of 11 species in 4 versions. The phase 1 version was selected for subsequent analysis.

A total of 24 scaffolds were assembled, encompassing 12 nuclear pseudo-chromosomes, two organelle genomes, and ten unplaced sequences. The nuclear chromosomes, designated as chr01 to chr12 based on their decreasing length, accounted for 99.71% of the total genome length, with a combined length of 740.32 Mb (**Figure 2**; **Supplementary Figure 1**). Single-ring molecules were identified in both mitochondria (483 kb) and chloroplasts (161 kb), referred to as Mt and Pt, respectively. Whole genome DNA sequencing yielded approximately 28 Gb of PacBio long reads (approximately 35× coverage), 71 Gb of Hi-C reads, and 90 Gb of Illumina short reads (approximately 100× coverage) (**Supplementary Table 1**). The PacBio long reads averaged 11 kb in length with an N50 value of 11 kb (**Supplementary Table 1**). The Hi-C reads exhibited Q20 and Q30 values of 68 Gb (96.6%) and 64 Gb (90.9%), respectively, while the Illumina short reads showed Q20 and Q30 values of 87 Gb (97.29%) and 83 Gb (92.91%) (**Supplementary Table 1**). Based on the newly generated data, a revised version of the chestnut genome was assembled (**Supplementary Table 2**). Initially, the primary genome assembly was generated by directly assembling PacBio long reads using hifiasm (Cheng et al., 2021). Subsequently, the Hi-C sequencing data were aligned to this primary assembly, enabling the reconstruction of each chromosome individually (**Supplementary Figure 2**). Finally, gaps were filled using HiFi ultra-long reads. Through rigorous manual sorting and redundancy removal, the final chestnut genome assembly was achieved. The genome size was determined to be 742.47 Mb, with a contig N50 length of 52.20 Mb, which represents a significant improvement in continuity compared to three previously published genomes (5.23 Mb, 2.83 Mb, 0.13 Mb, 0.94 Mb) (NC_GitHub, GWH, Hardwood_v4.3, GigaDb, cited) (**Table 1**). The final genome assembly contained only 13 unresolved gaps. The lengths of the three longest scaffolds were 90,863,751 bp, 90,442,070 bp, and 90,206,108 bp, respectively. Additionally, the average lengths of the scaffolds were 30,936,450 bp, 61,177,327 bp, 6,207,063 bp, and 102,176 bp, respectively (**Table 1**).

**Table 1:** Sequencing, assembly and annotation comparison among the eight Chinese chestnut.

	LYv1	GigaDb	YN	N11_1	Vanuxem	H7	YH	ZS
Sequencing								
Bases of WGS-long-read sequencing data (Gb)	28.6	69.31	100.6	66.98	*	95.01	99.22	83.62
Bases of WGS-Illumina (Gb)	89.141	35.44	80.8	54.55	*	101.44	110.32	86.98
Bases of Hi-C (Gb)	70.814	*	*	110,83	*	*	*	*
Bases of mRNA-seq (Gb)	52.908	20.84	*	10.3	*	*	*	*
Assembly								
Number of contigs	35	2,707	422	671	4,040	1,460	1,514	827
N50 of contigs (Mb)	47.97	0.94	5.88	0.0028	0.13	3.39	3.65	2.17
N90 of contigs (bp)	16,063,426	133,678	889,749	455.88	*	448,929	330,218	436,758
Number of scaffolds	24	*	*	112	*	*	*	*
N50 of scaffolds (Mb)	61.17	*	*	57,34	*	50.5	65.05	52.16
Final genome size (Mb)	726.73	785.53	734.13	688.93	412.79	671.99	790.99	678.9
GC content (%)	35.15	*	*	35.11	*	35.04	35.01	35
Complete BUSCOs (%)	95.6	96.7	97.4	92.44	94.1	90.97	90	95
Annotation								
Number of predicted protein-coding genes	35,008	36,479	45,011	33,074	20,376	32,411	31,792	32,012
Average gene length (Kb)	5.27	1,14	*	4,70	*	4.53	4.52	5.23
Repetitive sequences (bp)	395,599,549 (53.28%)	390,304,371	78,123,871 (59%)	366,839,546 (53.24%)	367,991,715 (50.74%)	442,757,988 (64.43%)	373,896,766 (54.99%)	423,161,924
		-49.69%						-53.49%
**Reference**	this study	Xing et al., 2019	Sun et al., 2020	Wang et al., 2020	Staton et al., 2020	Hu et al., 2022	Hu et al., 2022	Hu et al., 2022

Asterisk (*) indicates data were not shown in the original articles.

The quality of the chestnut genome assembly in this version was thoroughly evaluated. First, according to the Benchmarking Universal Single-Copy Orthologs (BUSCO) analysis, the assembly contained 1,376 (95.60%) complete or duplicated genes out of the 1,440 directly homologous single-copy genes, indicating a high level of completeness at the gene level (**Supplementary Table 3**). Secondly, the Long Terminal Repeat Assembly Index (LAI) value of the assembly was 16.52, which suggests substantial integrity at the transposon level. Additionally, 99.6% of the Illumina short reads aligned to the genome, and 99.7% of the total genome length was covered by PacBio long reads (**Supplementary Table 4**). Furthermore, the order of the chestnut genome chromosomes was generally consistent with that of the related *Quercus lobata* (**Supplementary Figure 3**) and two other published chromosome-level genomes, with the exception of the 3’ end of chr01, which was not assembled contiguously in other versions. In the NC_GitHub version, chromosome chr12 exhibited significant missing components (**Supplementary Figure 4**). In summary, the final genome assembly achieved the chromosome level with high continuity and integrity.

### Genome annotation

We predicted a total of 35,008 protein-coding genes, which accounted for 97.71% coverage of complete core eukaryotic BUSCO genes (**Supplementary Table 3**). Detailed annotations for these protein-coding genes are provided in **Supplementary Table 6**. The average lengths of the genes, exons, and CDS were calculated to be 5.27 kb, 0.32 kb, and 1.23 kb, respectively (**Supplementary Table 5**). Additionally, the analysis identified 670 tRNA genes, 1,923 rRNA genes, and 739 other non-coding RNA genes (**Supplementary Table 6**). Functions were predicted for 34,104 protein-coding genes (97.42%) based on sequence similarity and structural domains, while the functions of 904 genes remained unknown (**Supplementary Table 7**). The chestnut genome contained 53.28% (395.60 Mb) duplicated sequences, predominantly characterized by the highest content of LTR retrotransposons (LTR-RT), accounting for 28.08% (208.49 Mb) of the genome. This was followed by the DNA subfamily in DNA-like transposons, representing 19.35% (143.71 Mb) of the genome (**Supplementary Table 8**). Additionally, a category labeled as “Unknown” transposons accounted for 3.68% of the sequences. These unknown transposons represent elements that cannot be classified into known types, suggesting that various mutations accumulated over the process of evolution have rendered these transposons unclassifiable.

By comparing the numbers and lengths of different types of transposons, as well as their genome proportions and average lengths, among Fagaceae species (*Castanea mollissima*, *Quercus robur*, *Quercus fabri*, and *Quercus variabilis*), it was observed that LTR retrotransposons (LTR-RTs) exhibited greater diversity compared to DNA transposons. DNA transposons exhibited similar distribution patterns across all species. Class I transposons accounted for the largest proportion across the four species, with Gypsy being the most abundant. Within class II (DNA-type) transposons, the Helitron showed a higher proportion compared to other sub-classes (**Supplementary Table 8**). Overall, the characteristic patterns of transposons were consistent across the four species, although some species contained unique types not identified in others (**Supplementary Table 8**). For example, pararetrovirus transposons were exclusively found in chestnut (124 occurrences), and retrovirus transposons were uniquely identified in *Quercus* variabilis (54 occurrences) (**Supplementary Figure 5**; **Supplementary Table 8**). In the process of gene annotation, 35,008 non-redundant protein sequences were compiled from related species, such as *Quercus fabri*, *Quercus robur*, *Juglans regia*, *Corylus mandshurica*, and *Arabidopsis thaliana*, to provide evidence of protein homology. A total of 52.91 Gb of Illumina sequence data were collected from six different tissues (fruit, root, stem, tender leaf, bud, and bark) (**Supplementary Table 9**) This comprehensive dataset facilitated the prediction of 35,008 protein-coding genes, achieving a high level of completeness with a BUSCO score of 97.71%. The results revealed that the predicted protein-coding genes, which averaged 5.7 exons per gene, had an average length of 5,270.1 bp. Additionally, the average lengths of the coding sequences (CDS), exon sequences, and intron sequences were 1,231.1, 319.2, and 959.2 bp, respectively (**Supplementary Table 9**). From 18 samples, a total of 12.45 Gb clean reads were obtained, with an average yield of 3.71 Gb per sample (**Supplementary Table 10**). Alignment of these reads against the reference genomes demonstrated a total matched fragment alignment rate of 94.26% and an average mapping rate of 85.65% (**Supplementary Table 11**).

### Comparative genome and phylogenetic analysis

**Figure 3:**
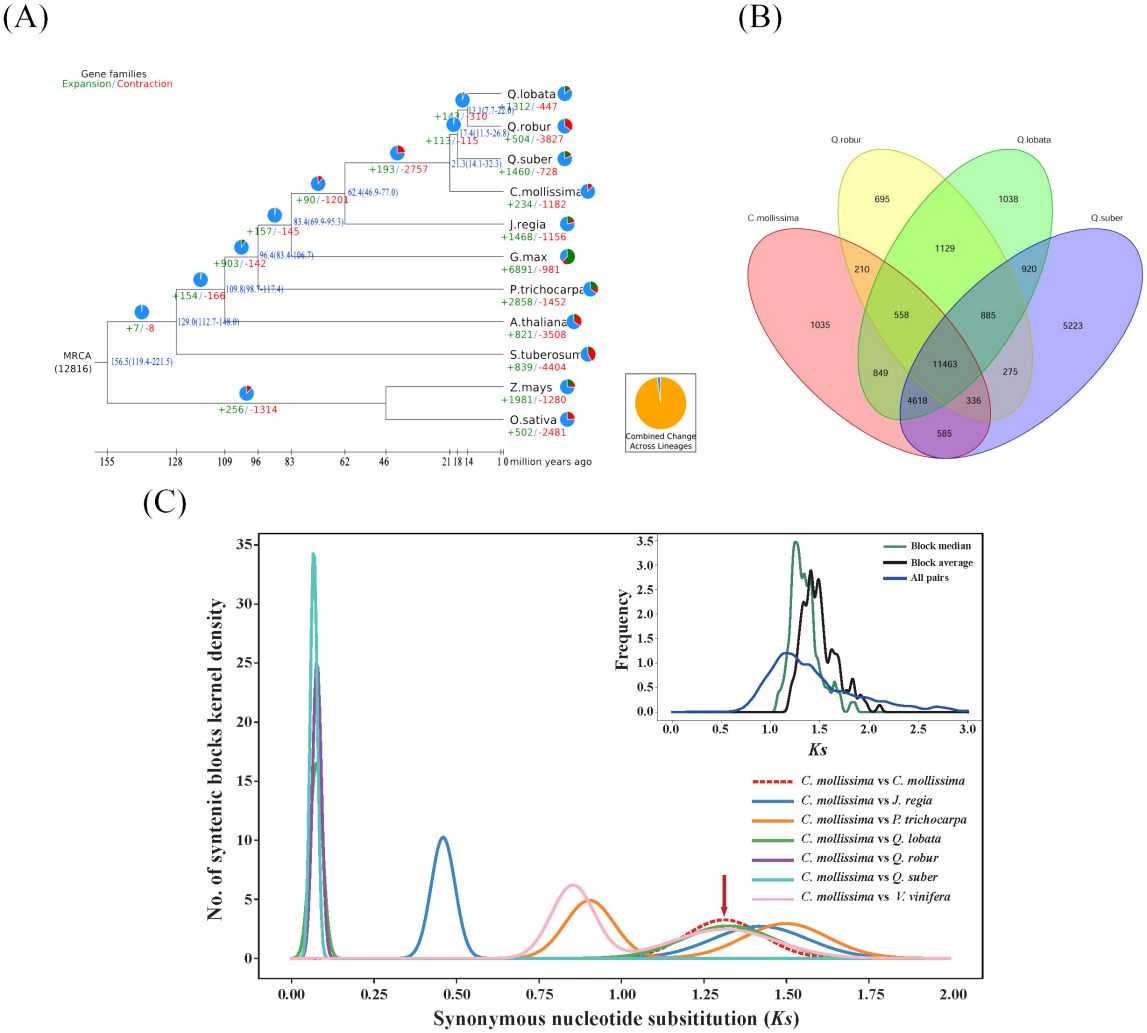
Genome evolution analysis **(A)**. Analysis on evolutionary history of Fagaceae genomes Timing diagrams showed differentiation time of Fagaceae, with node ages and 95% confidence intervals labeled. Pie diagrams showed proportions of gene families that underwent expansion or contraction; **(B)** Venn diagram of gene families of four Fagaceae species; **(C)** *K*s distribution map. Chestnut vs. chestnut (upper right); comparison between chestnut and other 6 species (*Juglans regia*, *Populus trichocarpa*, *Quercus fabri*, *Q. robur*, *Q. variabilis*, and *Vitis vinifera*).

The distribution of transposons within the chestnut genome demonstrated a preference for regions with high GC content, which are indicative of low gene density. Notably, Class I transposons was significantly enriched in these regions (**Figure 3A**). An analysis of 324 single-copy gene families, identified in chestnut and ten other plant species, was conducted. Full sequence alignment was performed using protein sequences to concatenate single-copy genes into a supergene. Codon positions 1, 2, 3, 1 + 2, and 4d were extracted. *Oryza sativa* and *Zea mays* were chosen as outgroups to construct evolutionary trees, labeled as phase0, phase1, phase2, and 4d, respectively (**Figure 3B**). The results from phase1, which demonstrated the highest confidence, were ultimately selected as the final phylogenetic tree and used for subsequent analyses. According to the phylogenetic tree, the divergence time between chestnut and three oak species (*Quercus robur*, *Quercus fabri*, and *Quercus variabilis*) was estimated to be approximately 21.3 million years ago, while the divergence from Oryza sativa (rice) and Zea mays (maize) was estimated to have occurred around 156.5 million years ago (**Figure 3A**).

An analysis was conducted to explore the characteristics of gene families in chestnut and ten other plant species. After filtering the data, a total of 335,835 genes were obtained, resulting in the identification of 46,626 gene families through clustering. Chestnut was found to have 19,654 gene families, a number generally consistent with those of the other ten plant species. In chestnut, 25,364 genes were identified, representing 72% of the total chestnut genes (**Supplementary Table 12**). Due to data filtering and the requirement that each gene family include at least two genes, 28% of chestnut genes were not clustered into gene families. Among the eleven plant species studied, 13.23% of the gene families (6,168 families) were shared, encompassing 32.44% of the genes (108,958 genes) (**Supplementary Table 7**). Notably, *Arabidopsis thaliana* exhibited the highest proportion of shared gene families at 42.50%, while *Quercus variabilis* had the lowest at 25.38%. In chestnut, the proportion of shared genes was 31.38%. Specific gene families unique to chestnut accounted for 4.0%, containing 1,794 genes (7.1%), which was notably less than that in *Quercus* variabilis but similar to those in *Quercus robur* and *Quercus fabri* (**Supplementary Table 12**). Compared to four closely related species within the Fagaceae family, a total of 29,820 gene families were identified, with 11,463 gene families being common and 1,035 gene families unique to chestnut. The number of unique gene families in chestnut was substantially smaller than that in *Quercus variabilis*, which had 5,223 unique gene families. (**Figure 3B**).

The *K*s (Substitutions per synonymous site) value between two homologous genes reflected the extent of sequence conservation from the time of replication or divergence to the present. It represents the evolutionary status of the gene family, increasing over time due to cumulative mutations driven by natural selection. In this study, it was observed that the ancestors of chestnut underwent early replication events, similar to other species, but not in the recent period (**Figure 3C**). Both chestnut and *Quercus variabilis* exhibited peaks around *K*s = 0.06, representing the divergence time of the two species, estimated to be approximately 3.9 million years ago (**Figure 3A**). Using Mega 7.0 to calculate the relative synonymous codon usage (RSCU) values, the formula 0.06/2/(6.5*10^-9) yields an estimated divergence time of 4.6 million years ago, which aligns with the differentiation time.

### Expansion of the sugar metabolism gene family

**Figure 4:**
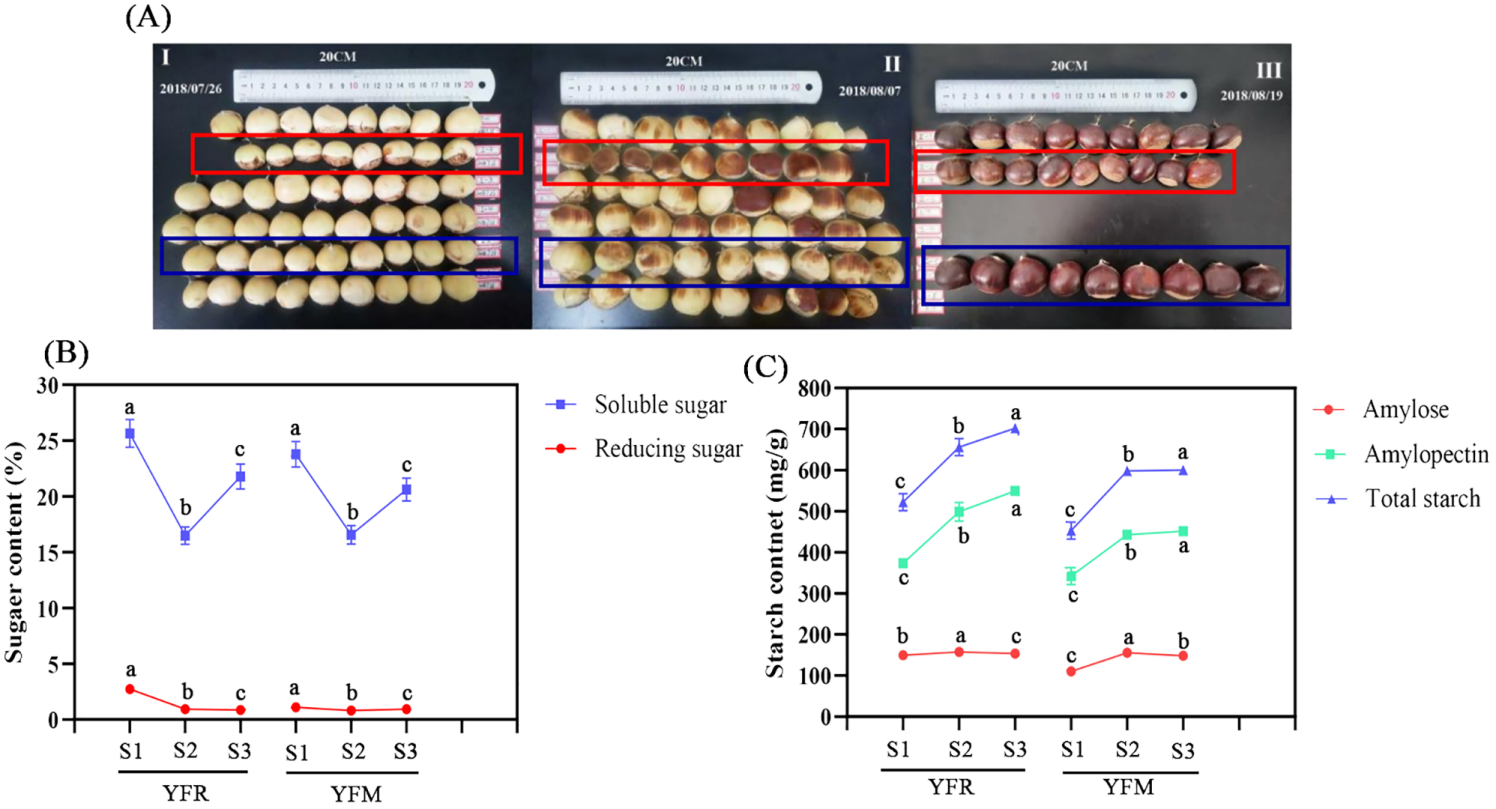
Content changes of starch and sugar in chestnut seed kernels in different time series. **(A)** Fruit growth status of F1 chestnuts from different male parents. **(B)** Sugar content; **(C)** Starch content. YFR: Yongfeng 1♀ × Yongren Zao♂. YFM: Yongfeng 1♀ × Yimen 1♂. S1-S3: three time points before chestnuts mature: 20 days, 10 days, and 0 days. a-c: There is a significant difference at the p<0.05 level.

The expansion and contraction characteristics of the gene families in chestnut and ten other plant species were analyzed based on the gene family data. The results revealed substantial variability in the patterns of gene family expansion and contraction among these species. The number of expanding gene families ranged from 234 (1.2%) in chestnut to 6,891 (39.37%) in *Glycine max*. Conversely, the number of contracting gene families ranged from 447 (2.1%) in *Quercus fabri* to 4,404 (29.6%) in *Solanum tuberosum* (**Figure 4A**; **Supplementary Table 12**). These variations can be attributed to genome-wide duplication events, differences in gene numbers, and the phylogenetic relationships among species.

In chestnut, we identified234 (1.2%) expanding gene families and 1,182 contraction gene families (**Supplementary Table 13**). This pattern of significant contraction is similar to that observed in the related species *Quercus robur*. In contrast, other related species *Quercus fabri* and *Quercus variabilis* showed notable expansion in their gene families. KEGG enrichment analysis revealed that genes in the expansion gene families were significantly enriched in several biosynthetic pathways (**Supplementary Figure 6**): (1) biosynthesis pathways such as monoterpenoid biosynthesis and phenylpropanoid biosynthesis; and (2) sugar metabolism pathways, including starch and sucrose metabolism, as well as amino sugar and nucleotide sugar metabolism. On the other hand, genes in the contraction gene families were significantly enriched in different pathways (**Supplementary Figure 7**): (1) biosynthesis pathways such as isoquinoline alkaloid biosynthesis and phenylpropanoid biosynthesis; and (2) secondary metabolism pathways, such as linoleic acid metabolism and tyrosine metabolism.

### Starch biosynthesis pathway and gene annotation in chestnut

Three biological replicates of seed kernel tissues were collected at each time point for the measurement of sugars and starch, resulting in a total of six metabolites measured. The content of all measured metabolites exhibited a progressive increase over time. Notably, in both YFR and YFM samples, the content of soluble sugars was significantly higher than that of reducing sugar, while the levels of amylopectin exceeded those of amylose (**Figures 4A, B**).

**Figure 5:**
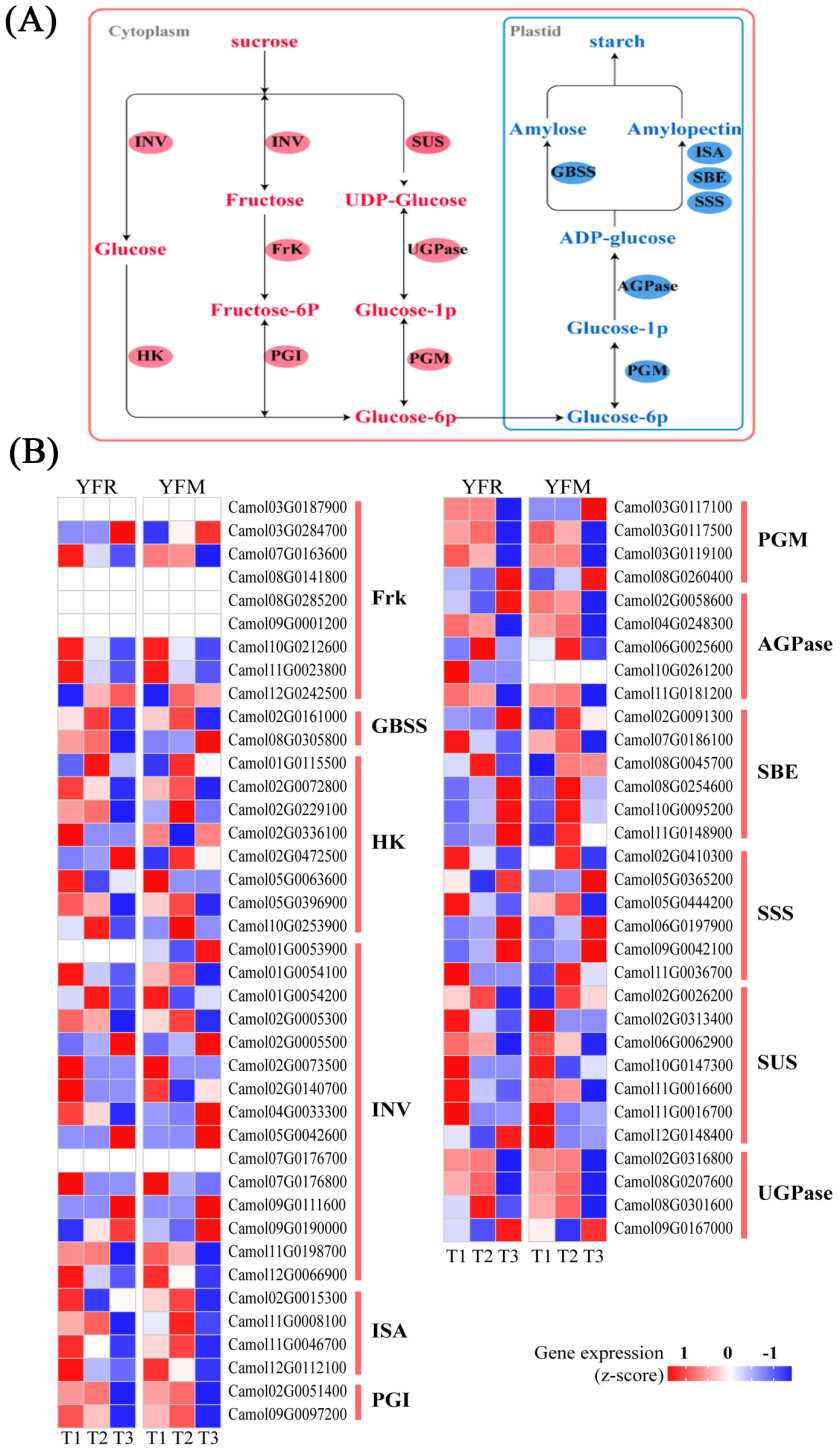
Starch metabolism pathways of chestnut transcriptome. **(A)** Starch and sugar metabolism pathways. AGPase, adenosine diphosphate-glucose pyrophosphorylase; GBSS, granule-bound starch synthase; SSS, starch synthases; GBE, glucan-branching enzymes/starch branching; SUS, sucrose synthase; HK, Hhexokinase; FrK, fructokinase; INV, invertase; PGI, Phosphoglucose Isomerase; PGM, phosphoglucomutase. **(B)** Heat map of related gene expression in starch metabolism pathways of chestnut transcriptome.

The gene family responsible for encoding the enzyme INV (invertase) in the sucrose hydrolysis pathway was found to possess the largest number of structural genes, with a total of 15 members identified (**Figure 5**). In the starch biosynthesis pathway, ADP-glucose pyrophosphorylase (AGPase) was identified as the initial rate-limiting enzyme, and five AGPase-encoding genes were discovered. Interestingly, one of these genes (*Camol02G0058600*) exhibited significant expression specifically in the YFR hybrid combination (**Figure 5**). The enzymes granule-bound starch synthase (GBSS), starch synthase (SSS), starch branching enzyme (SBE), and debranching enzyme (ISA) are responsible for converting ADP-glucose to starch. In the YFR hybrid combination, four *SBE* genes (*Camol02G0091300*, *Camol08G0254600*, *Camol10G0095200*, and *Camol11G0148900*) and three *SSS* genes (*Camol05G0365200*, *Camol06G0197900*, and *Camol09G0042100*) exhibited remarkably high expression levels at T3, with the four SBE genes being exclusively highly expressed in the YFR hybrid combination (**Figure 5**). These findings indicate that the starch biosynthesis genes identified in this study displayed distinctive high expression patterns at the T3 stage of the YFR hybrid combination, suggesting that they may play a crucial role in the high starch content observed in this particular combination. Furthermore, a total of 1,533 transcription factors belonging to 58 families were annotated, with the MYB family exhibiting the highest number of members (142).

### Construction of temporal gene co-expression network

**Figure 6:**
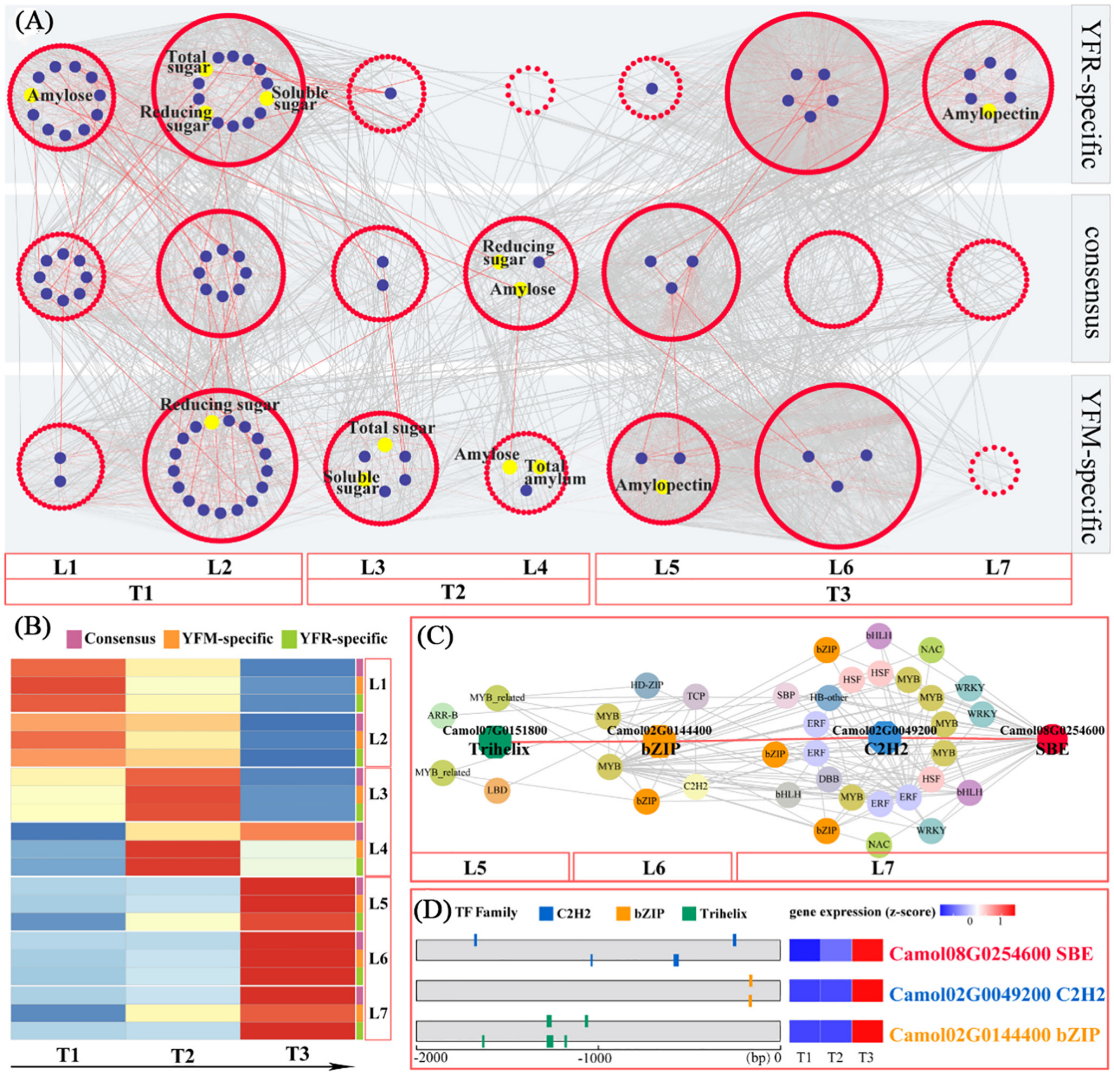
Temporal gene regulation of chestnut starch biosynthesis. **(A)** Predicted regulatory network and relationships between TFs and enzyme genes involved in starch biosynthetic pathway (enzymes are shown by blue dots; TFs are shown by red nodes); L1 to L7 represent levels found in three temporal gene co-expression networks (YFR-specific, YFM-specific and GCN-consensus). **(B)** Heatmap of average TPM (z-score normalization) for each level of TO-GCN at every time point; based on expression profiles, three sub-networks of starch biosynthesis were identified, which were T1 (L1-L2), T2 (L3-L4) and T3 (L5-L7). **(C)** Grading rules for SBE central genes. **(D)** Gene expression (TPM) and TF binding site (TFBS) were detected in the 2kb upstream sequence of central gene (SBE), and three potential regulator TFs were detected; bZIP and C2H2.

The temporal gene co-expression network associated with starch synthesis was divided into seven classes, which were further organized into three sub-networks: YR-specific, YM-specific, and consensus networks. These sub-networks comprised 2, 6, and 5 content indicators; 22, 28, and 33 starch synthesis-related genes and 560, 649, and 627 transcription factors in the consensus, YFM, and YFR-specific networks, respectively (**Supplementary Table 14**). Based on the heat map, the seven levels could be further categorized the seven levels into three stages: Level 1-2 corresponding to the T1 stage, Level 3-4 corresponding to T2 stage, and Level 5-7 corresponding to T3 stage (**Supplementary Table 15**). A comprehensive transcriptional regulatory network was constructed, consisting of seven levels (L1-L7) and encompassing consensus, YFM-specific, and YFR-specific networks (**Supplementary Table 15**). Based on distinct expression patterns, these networks were further divided into three sub-networks: the T1 sub-network (L1-L2), which represents the starch accumulation at time point T1; the T2 sub-network (L3-L4), which corresponds to the starch accumulation at time point T2, and the T3 sub-network (L5-L7), representing the starch accumulation at time point T3 (**Supplementary Table 15**). A total of 560 transcription factors and 22 structural genes were consistently present across both YFM and YFR (**Figure 6A**), indicating that these two F1 hybrids exhibit a consistent pattern of co-expression regulation in starch accumulation. Notably, the T1 and T2 sub-networks contained a higher number of structural genes (19 members), showing consistent regulatory patterns and an association with sugar biosynthesis. This indicates that YFR and YFM have conserved gene co-expression networks. Furthermore, the bHLH transcription factor family played a prominent role in both the T1 and T2 sub-networks, encompassing 20 and 11 members, respectively (**Figure 6B**; **Supplementary Table 14**).

Among the sub-networks corresponding to the three periods, the T3 sub-network had the highest number of gene members, with 244, 301, and 340 members, respectively. The T1 sub-network followed with 183, 237, and 268 genes, while the T2 sub-network contained the fewest gene members, comprising 157, 145, and 57 members, respectively. The overall consensus network was comparable in size to the specific network, suggesting that the gene co-expression regulation patterns in YR and YM were largely consistent. Within the consensus network, members from various gene families predominantly contributed to the construction of the co-expression network. Each sub-network was characterized by certain transcription factor families. The T1 sub-network was dominated by the bHLH, GRAS, C2H2, and B3 families, comprising 20, 15, 12, and 11 members, respectively. The T2 sub-network was mainly characterized by the ERF, MYB, and bHLH families, with 13, 13, and 11 members, respectively. In the T3 sub-network, the MYB, ERF, WRKY, and bHLH families were predominant, with 30, 29, 21, and 18 members, respectively, indicating the crucial roles that these transcription factor families play in the co-expression network associated with sugar-starch synthesis.

### Key genes involved in starch and sugar pathways

The gene co-expression patterns of the two hybrid combinations, YFM and YFR, demonstrated distinct differences in sugar and starch content. For instance, reducing sugar and amylose content were exclusively identified in the T2 sub-network of the consensus network (L4). Conversely, amylopectin_content was located in the T3 sub-network of the YR-specific network (L7). In L7, five structural genes related to starch synthesis were identified, including two SBEs genes (*Camol02G0091300* and *Camol08G0254600*) and one *AGPase* gene (*Camol02G0058600*), which are significant genes encoding rate-limiting enzymes in the starch biosynthesis pathway. Based on their connectivity, the hub gene (*SBE*; *Camol08G0254600*) with the highest internal connectivity was selected as the target for constructing a hierarchical regulatory network (**Figure 6C**). Validation of transcription factor binding sites revealed a hierarchical regulatory cascade, where the *Trihelix* transcription factor (*Camol07G0151800*) regulates the bZIP transcription factor (Camol02G0144400), which in turn regulates the *C2H2* transcription factor (*Camol02G0049200*) and the *SBE* gene (*Camol08G0254600*).

In the T3 sub-network of the YFR-specific TO-GCN, the chestnut pollen texture showed a significant association with amylopectin formation (**Figure 6C**). Within this sub-network, the structural gene *SBE* (*Camol08G0254600*) was identified as the central hub gene with the highest connectivity (**Figure 6C**). *SBE* encodes a key rate-limiting enzyme in the amylopectin biosynthetic pathway. Through hierarchical regulatory network analysis, it was revealed that *Trihelix* (*Camol07G0151800*) likely functions as the tertiary regulator, *bZIP* (*Camol02G0144400*) serve as an intermediate regulator, and *C2H2* (*Camol02G0049200*) act as the primary direct regulator of SBE (*Camol08G0254600*) (**Figure 6D**).

## Discussion

The newly assembled telomere-to-telomere (T2T) gapless genome of the Chinese chestnut exhibits exceptional continuity, reliability, and completeness, representing the highest quality version of the Chinese chestnut genome published to date. This high-quality genome assembly significantly enhances the genomic resources available for *Castanea* species and has facilitated a comprehensive identification of transposable elements within the Fagaceae family, including chestnut, *Quercus robur*, *Q. alba*, and *Q. variabilis*. Comparative genomic analysis among chestnut and its closely related species revealed that chestnut genes account for 31.28% of the shared gene families, whereas *Q. variabilis* genes comprise only 25.38%.However, the Chinese chestnut has 793 gene families (4.0%), including 1,794 genes (7.1%), which is substantially lower than those in *Q. variabilis* (Sun et al., 2020). Additionally, comparative analysis of four closely related species within the Fagaceae family identified 29,820 gene families, of which 11,463 were shared among all species. There were 1,035 gene families unique to chestnut, significantly fewer than the 5,223 unique gene families found in *Q. variabilis* (Wang et al., 2020). Similar to its closely related species *Q. robur*, chestnut has exhibited a significant contraction in gene families (Staton et al., 2020). In contrast, *Q. alba* and *Q. variabilis* have shown significant expansion in their gene families. Previous studies have demonstrated that duplicated sequences in mitochondria genome play key roles in intermolecular recombination (Dong et al., 2020). The Chinese chestnut is rich in duplicated sequences, suggesting that it may have frequently undergone intermolecular recombination during its evolution, thereby altering its genome sequences and conformation.

The study of pollen xenosis holds significant importance for fruit production, providing a theoretical basis for the selection of pollination varieties, improvement of fruit quality, and enhancement of economic benefits. Carbohydrate content is an important indicator of pollen sensitivity of pollen xenosis. However, the molecular mechanisms underlying the relationship between carbohydrate metabolism and pollen xenosis remain largely unexplored. Starch serves as a crucial storage form of carbohydrates in chestnut kernels, while sucrose functions primarily as the main form of sugar transport. Both sucrose and starch metabolism are vital for the growth and development of chestnut kernels (Chen et al., 2017). Due to pollen xenia, the starch content in the YFR hybrid combination was significantly higher than that in the YFM hybrid combination, particularly with respect to amylopectin. AGPase, GBSS, SSs, SBEs, and ISAs have been identified as key enzymes for starch biosynthesis (Smith and Zeeman, 2020). We identified 23 genes encoding major enzymes for starch biosynthesis, including *AGPase* (5 genes), *GBSS* (2 genes), *SSS* (6 genes), *GBE* (6 genes), and *ISA* (4 genes). Analysis of gene co-expression patterns revealed distinct trends in sugar and starch contents between the two F1 pollinated species (YFR and YFM). Notably, only reducing sugars and starch were associated with the T2 sub-network (L4) of the consensus network. Conversely, YFR branched starch was categorized into L7 of the YR-specific T3 sub-network. Within this L7, there are 5 starch synthesis-related structural genes, including two *SBEs* (*Camol02G0091300* and *Camol08G0254600*) and one *AGPase* (*Camol02G0058600*), which are all important rate-limiting enzyme-encoding genes in the starch biosynthesis pathway.

The pathway of starch biosynthesis is initiated by the synthesis of the glucose donor adenosine diphosphate glucose (ADP-glucose) through the action of adenosine diphosphate glucose pyrophosphorylase (AGPase), which plays a critical role in regulating carbon flux into starch. Previous studies have demonstrated that AGPase significantly influences this carbon flux under non-stress conditions in Arabidopsis (Zhu et al., 2020) and potato tubers (Geigenberger et al., 2004). In the amylose region of amylopectin, the 1,4-α-glucan branching enzyme (SBE) hydrolyzes α-1,4 glycosidic bonds and subsequently reconnects them by forming new α-1,6 glycosidic bonds (Pfister et al., 2016; Ren et al., 2017). Additionally, isoamylase (ISA) is a starch debranching enzyme that specifically hydrolyzes α-1,6 glycosidic bonds, thereby regulating branching and maintaining the structural integrity of amylopectin granules. Plants possess multiple debranching enzyme genes, some of which are involved in amylopectin biosynthesis, while others play a role in starch degradation. The necessity of debranching for normal amylopectin biosynthesis has been established through studies of cereal mutants as well as experiments involving Chlamydomonas, Arabidopsis, and potato, all of which lack ISA1-type isoforms. This deficiency results in an increased branching frequency of glucans, a significant portion of which fails to form granules and remains soluble (Zhu et al., 2019).

Overall, the pollen xenia effect in chestnuts has been systematically investigated for the first time. Our findings revealed significant pollen xenia effects on the sugar and starch content of chestnut kernels across various developmental stages. To promote early fruiting, improve chestnut flavor, and enhance nut quality and economic returns, ‘Yongren Zao’ and ‘Yanlong’ are recommended as pollination varieties. Furthermore, the integration of genomic and transcriptomic data has elucidated the molecular regulatory mechanisms underlying the pollen xenia effect on starch accumulation in chestnuts. This research provides a critical foundation for future studies in chestnut population genetics, genetic improvement, and strategies aimed at increasing chestnut yield, quality, and economic benefits.

## Data Availability

The original contributions presented in the study are publicly available. This data can be found at the National Center for Biotechnology Information (NCBI) using accession number PRJNA912750.

## Supplementary material

Supplementary Figure 1 The position of telomere on 12 chromosomes of Chinese chestnut.

Supplementary Figure 2 Whole genome analysis on chromatin interactions based on Hi-C data

Supplementary Figure 3 Collinearity of *Castanea mollissima* with *Quercus lobata* (top left) and published versions (top right: NC_GitHub; bottom left: GWH). The horizontal axis is the *C. mollissima* assembly.

Supplementary Figure 4 Collinearity analysis on chestnut genome and *Quercus robur*, *Vitis vinifera* genomes.

Supplementary Figure 5 Comparison on TE between chestnut and other three Fagaceae species.

Supplementary Figure 6 KEGG enrichment analysis on genes in contraction gene families.

Supplementary Figure 7 Classification and statistics on duplication patterns of chestnut genes.
