# Supplementary figures and images for: Whole genome sequencing of Castanea mollissima and molecular mechanisms of sugar and starch synthesis
Source: Front Plant Sci. 2024 Oct 25;15:1455885. doi: 10.3389/fpls.2024.1455885 (PMC11543529; doi:10.3389/fpls.2024.1455885)

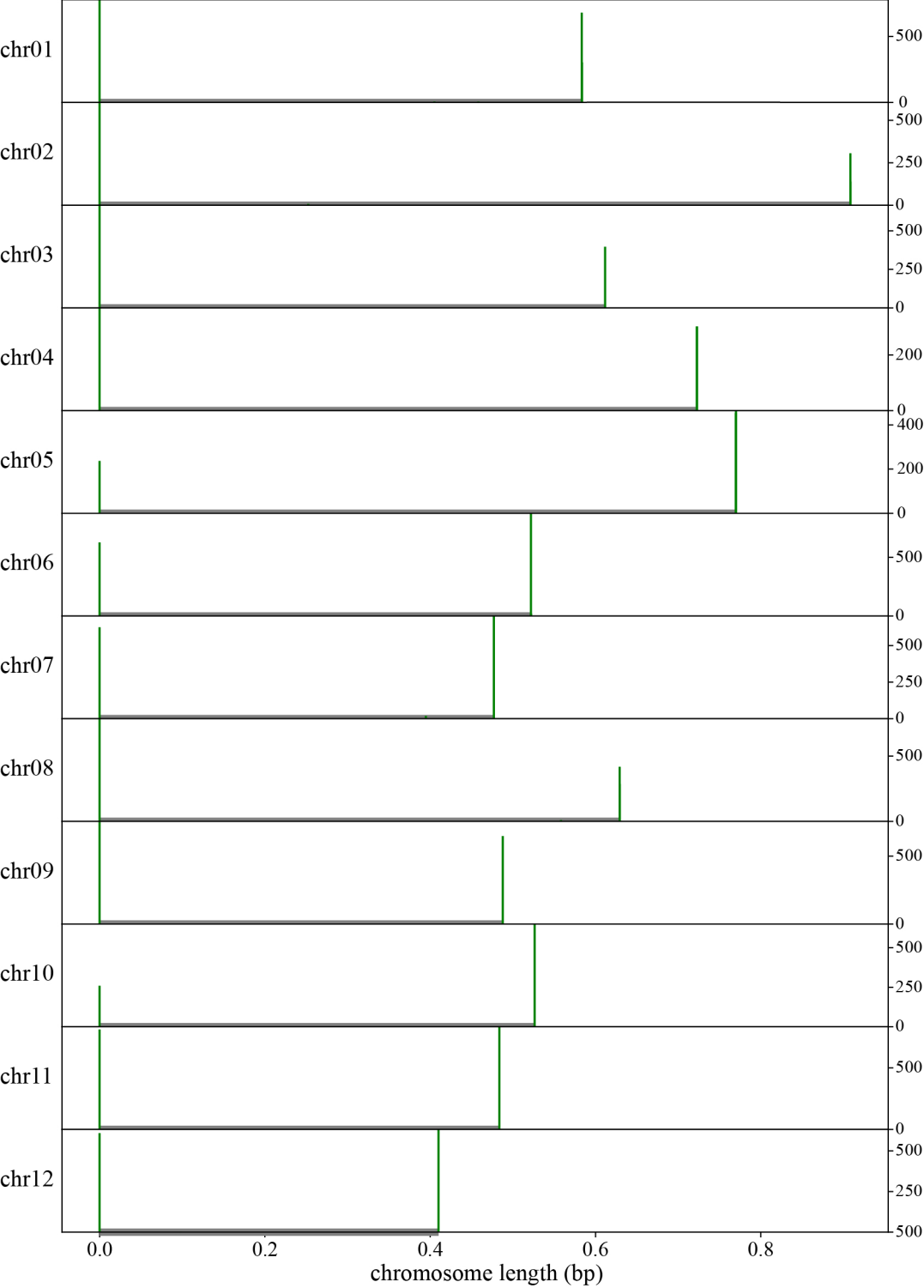

Supplement: Supplementary Figure 1 — The position of telomere on 12 chromosomes of Chinese chestnut. [file DataSheet1.zip › Figure S1.pdf]

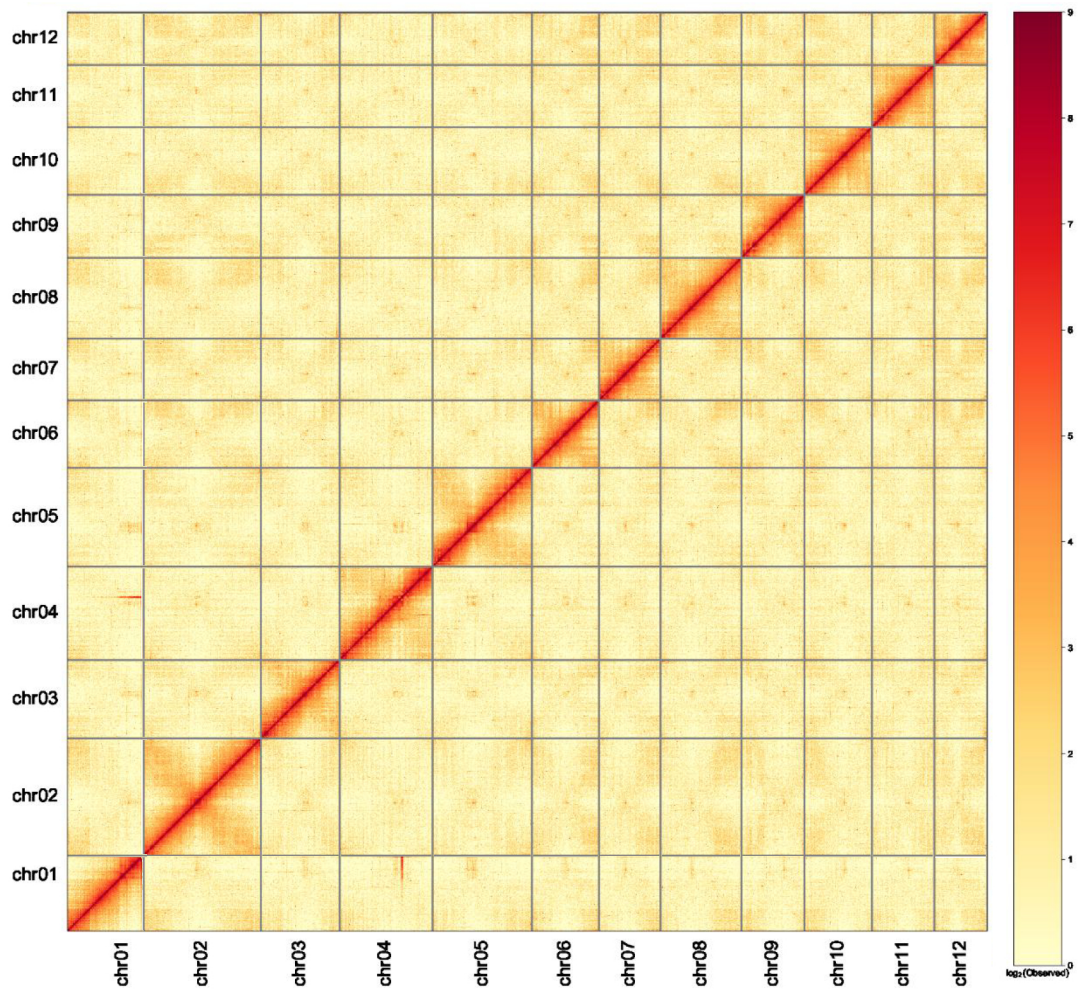

Supplement: Supplementary Figure 1 — The position of telomere on 12 chromosomes of Chinese chestnut. [file DataSheet1.zip › Figure S2.pdf]

(A)

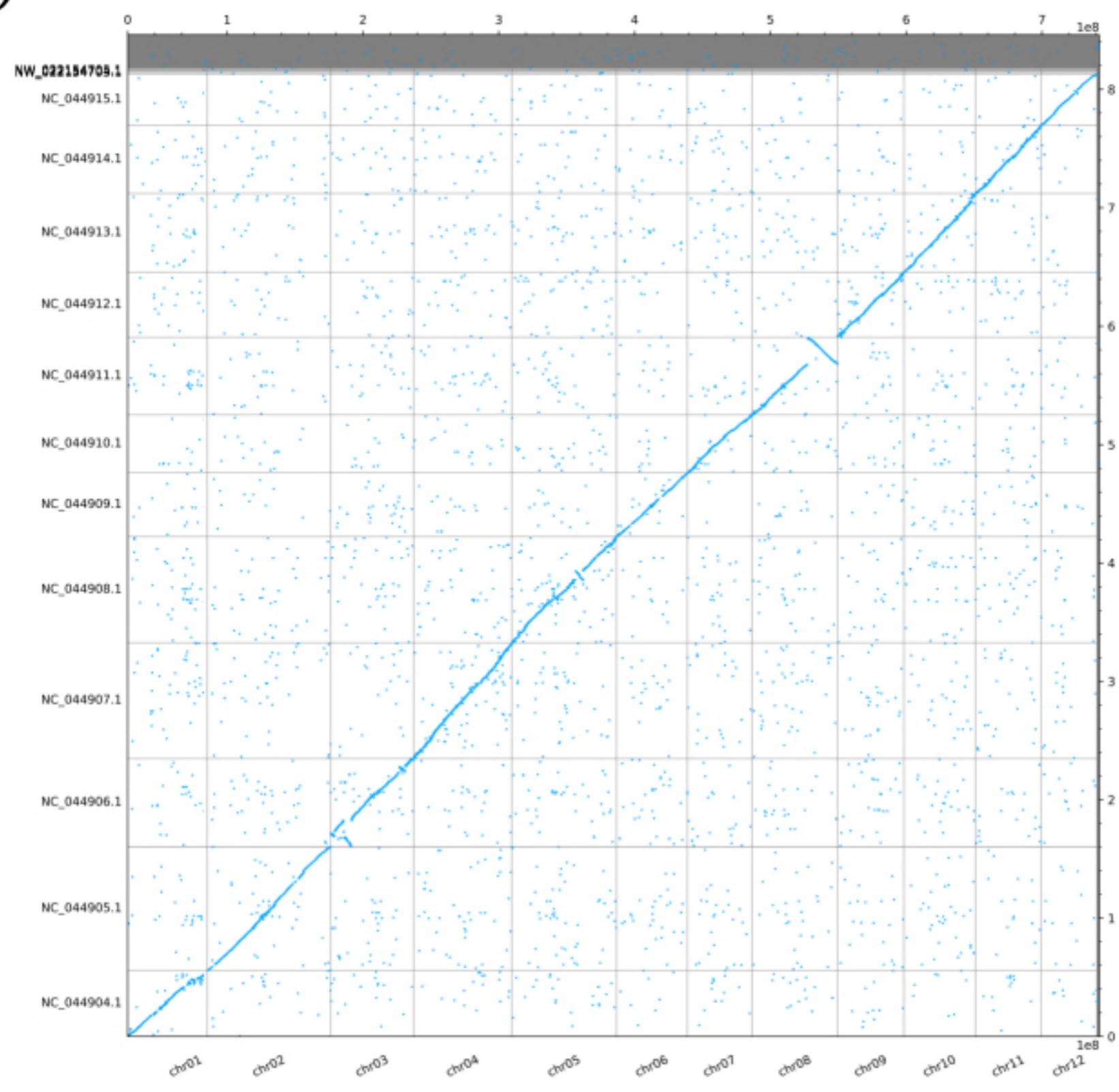

(B)

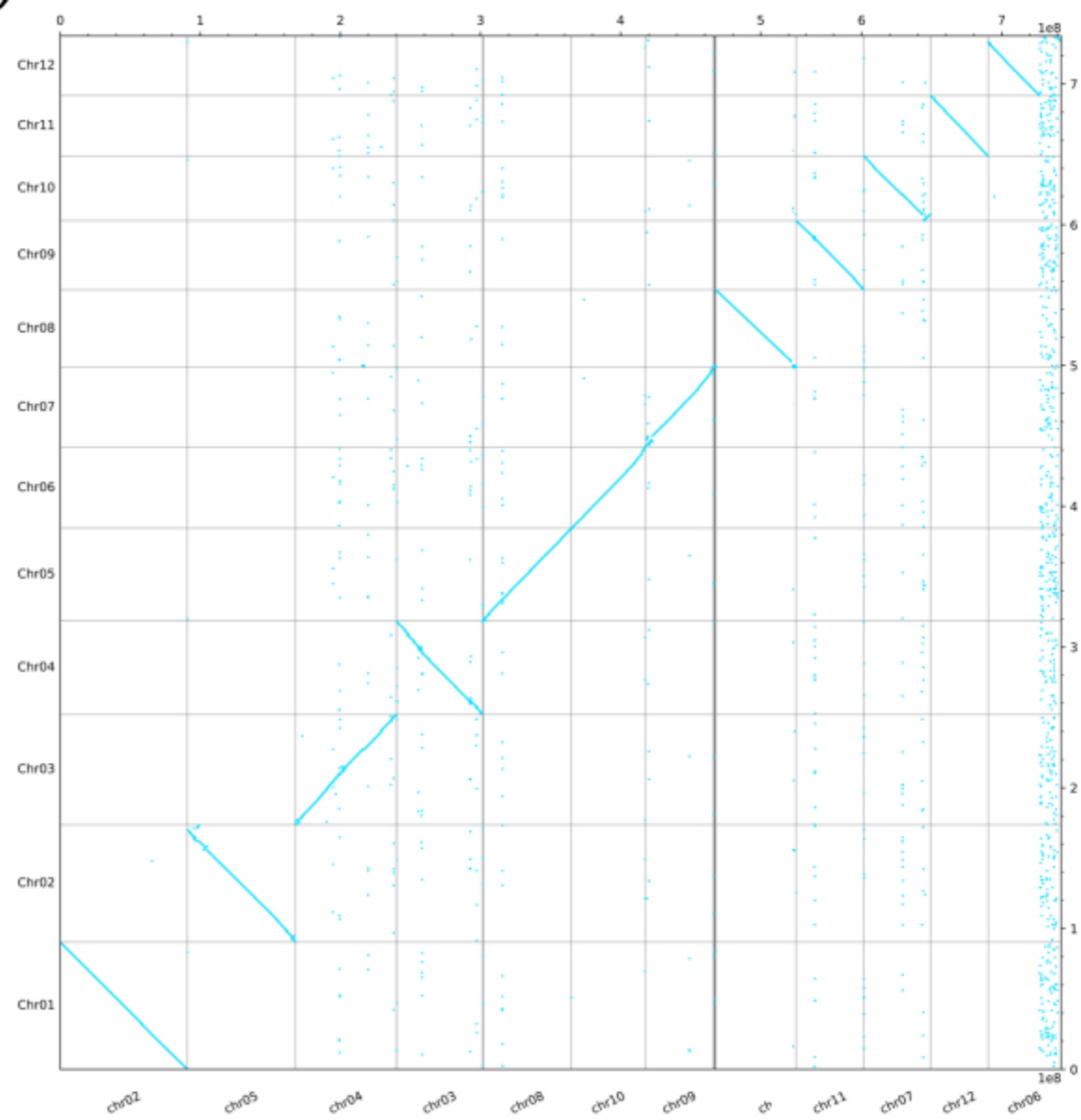

(C)

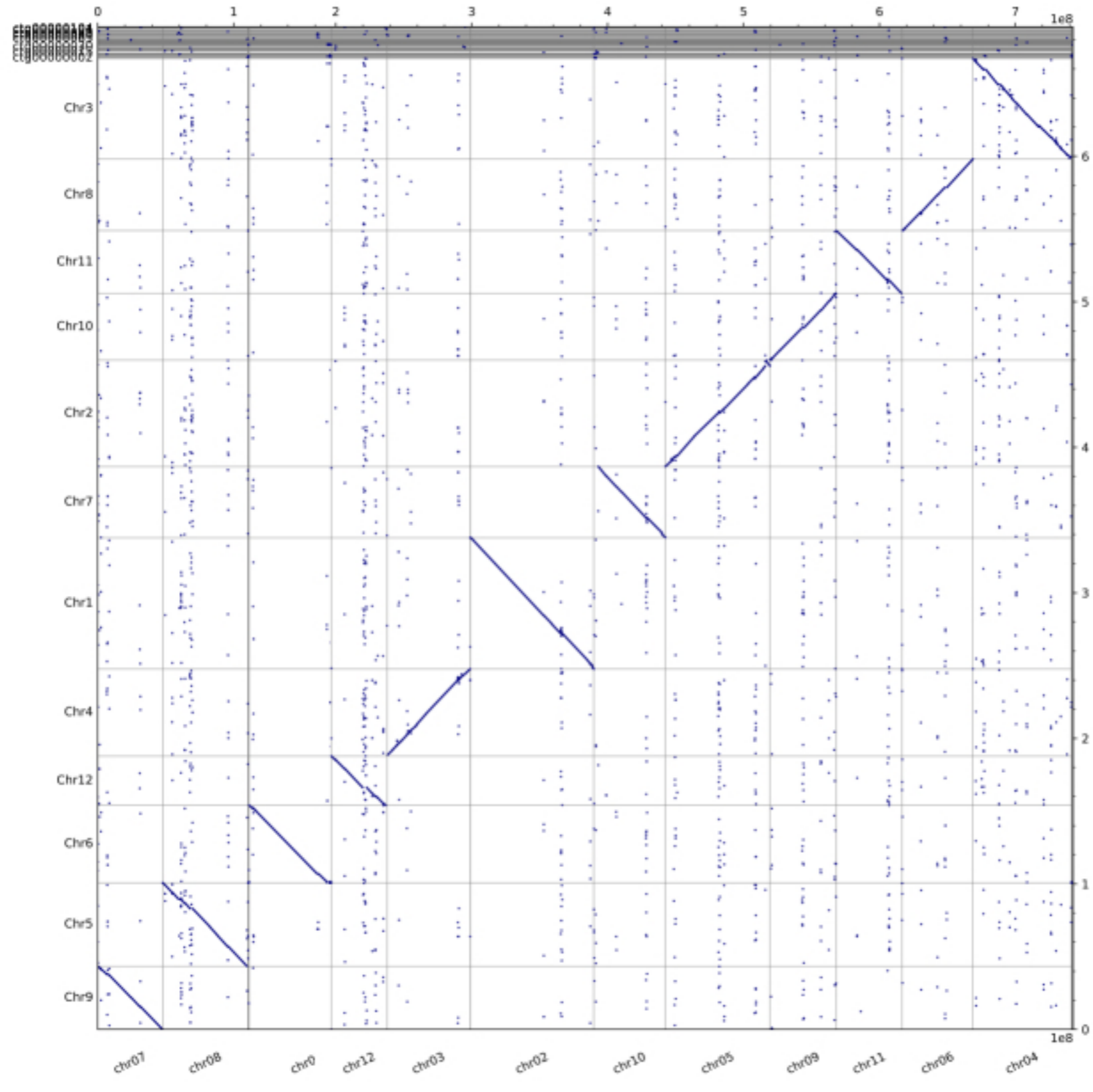

Supplement: Supplementary Figure 1 — The position of telomere on 12 chromosomes of Chinese chestnut. [file DataSheet1.zip › Figure S3.pdf]

(A)

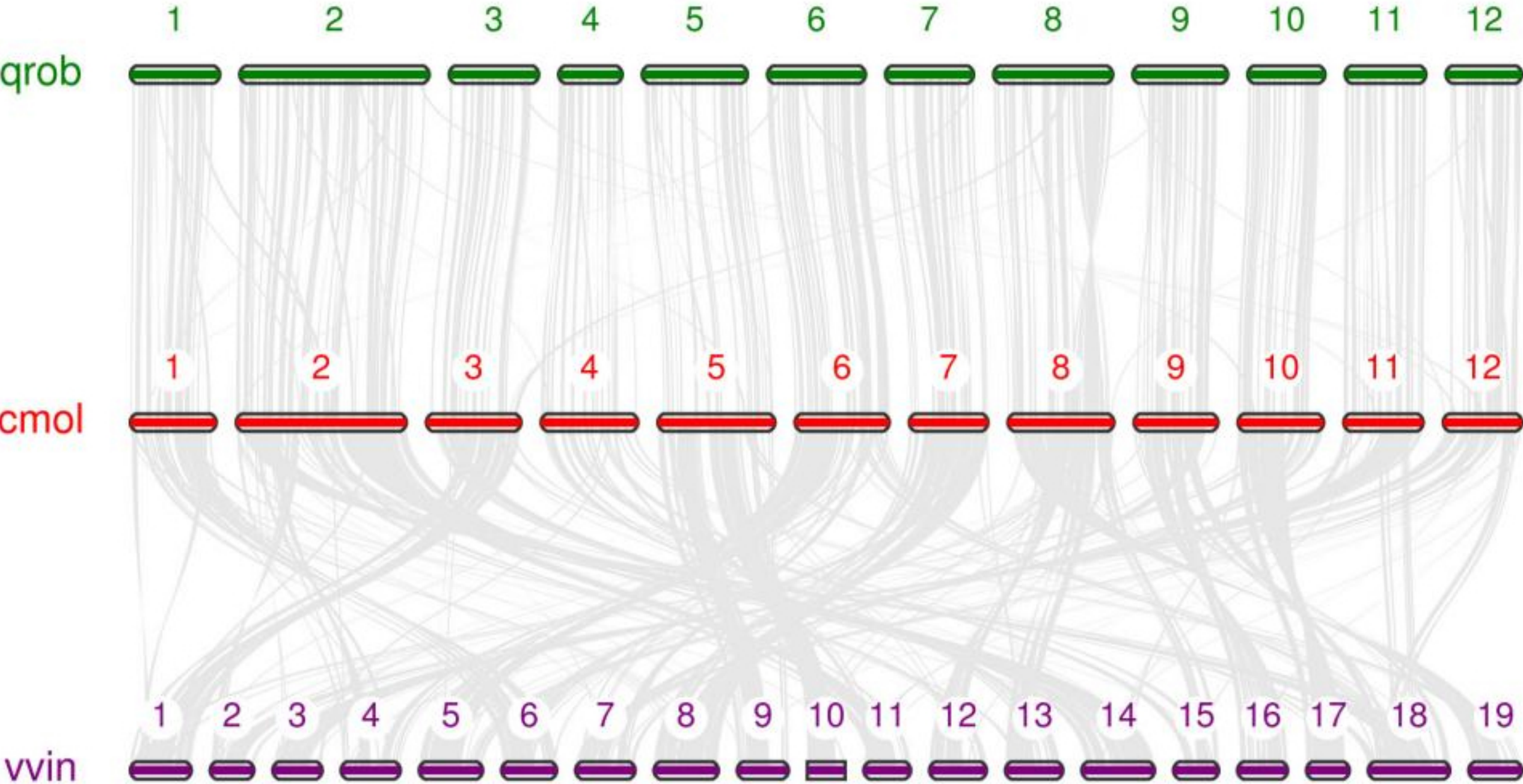

(B)

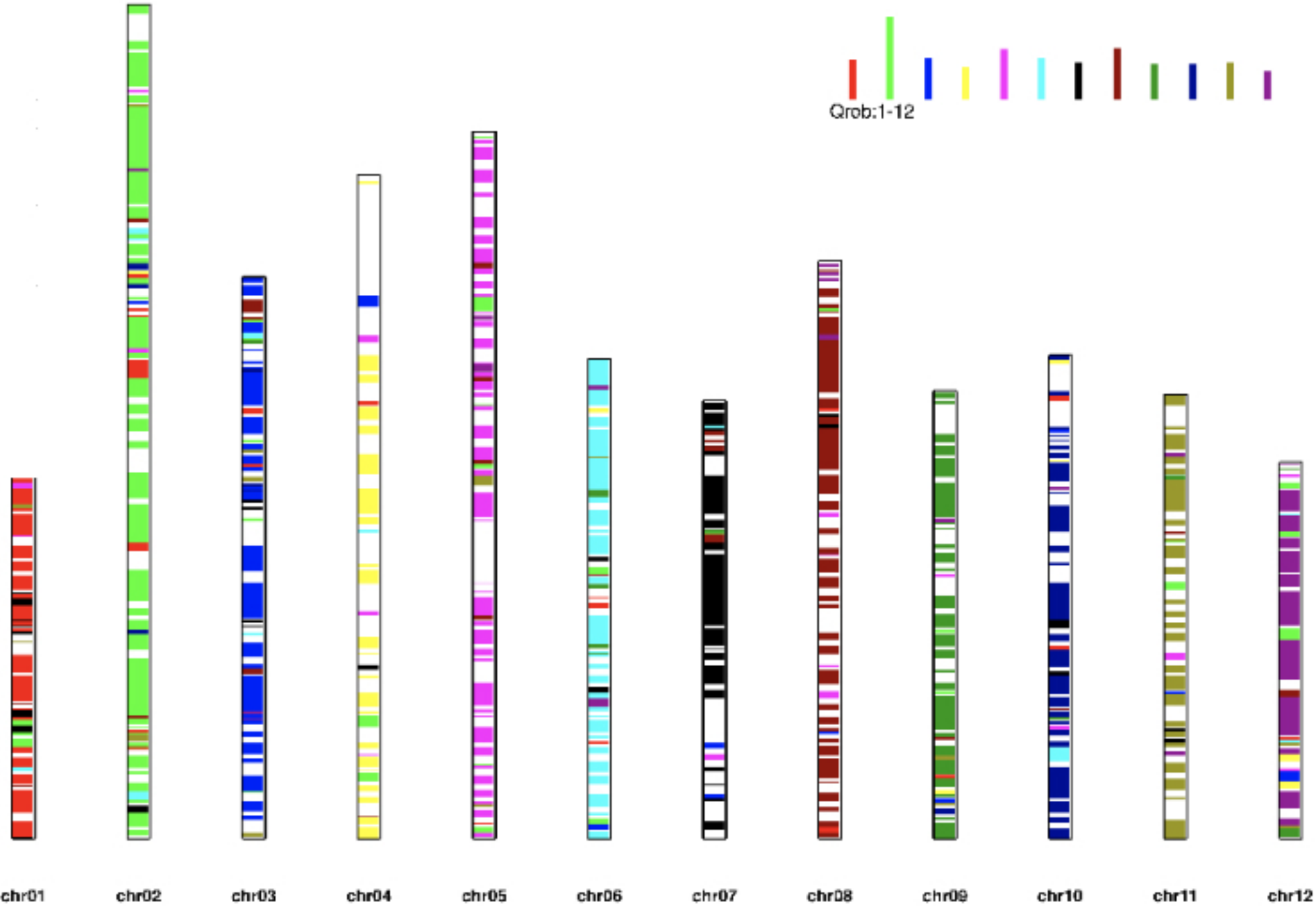

(C)

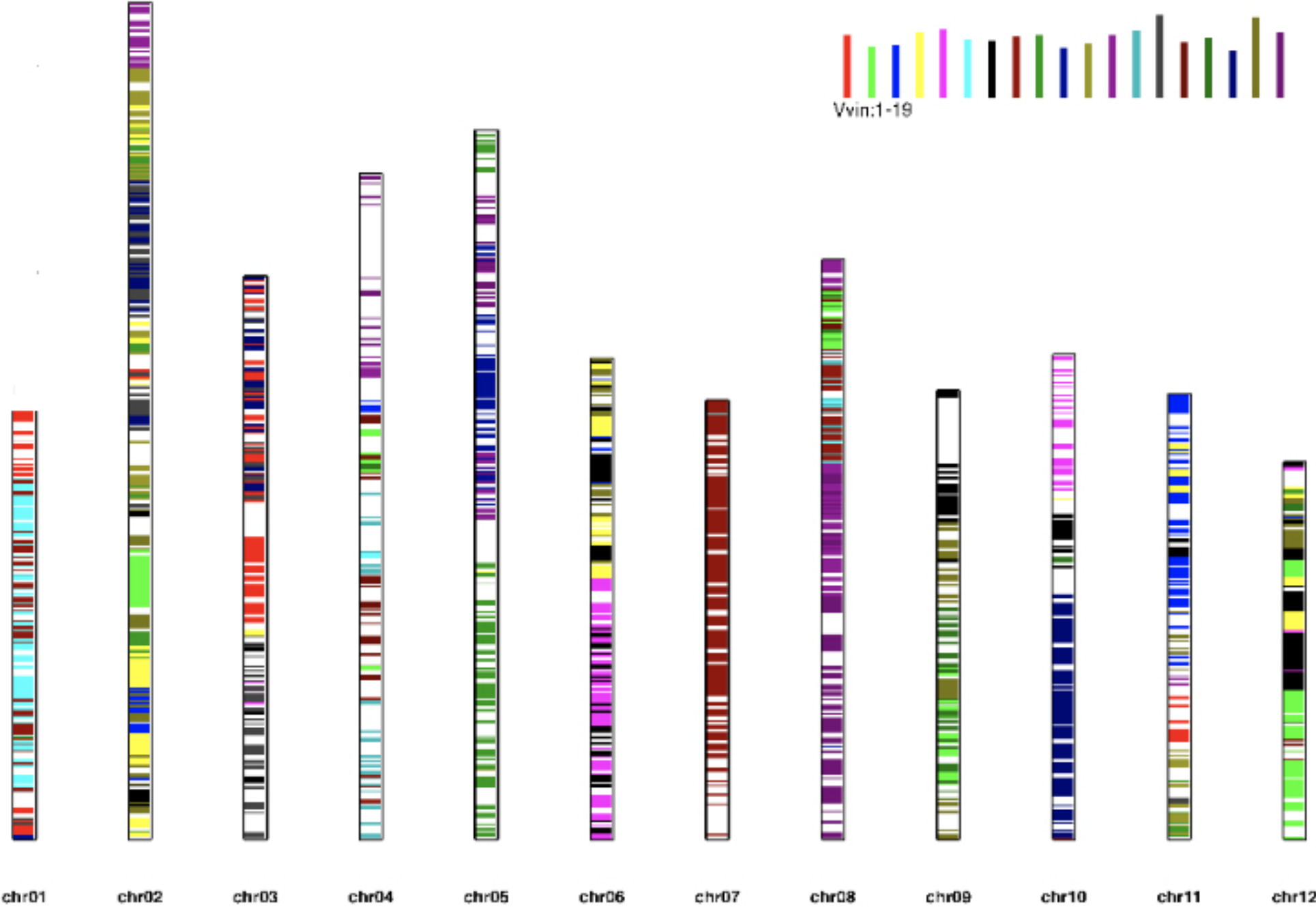

Supplement: Supplementary Figure 1 — The position of telomere on 12 chromosomes of Chinese chestnut. [file DataSheet1.zip › Figure S4.pdf]

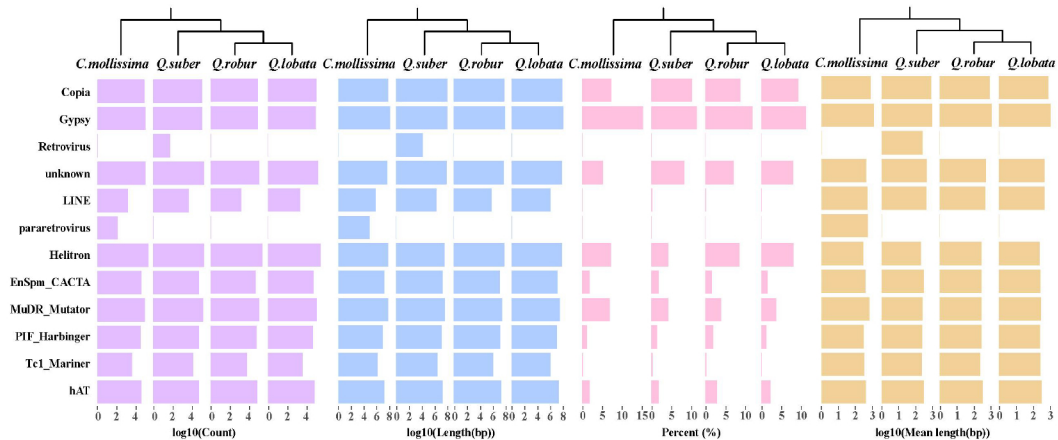

Supplement: Supplementary Figure 1 — The position of telomere on 12 chromosomes of Chinese chestnut. [file DataSheet1.zip › Figure S5.pdf]

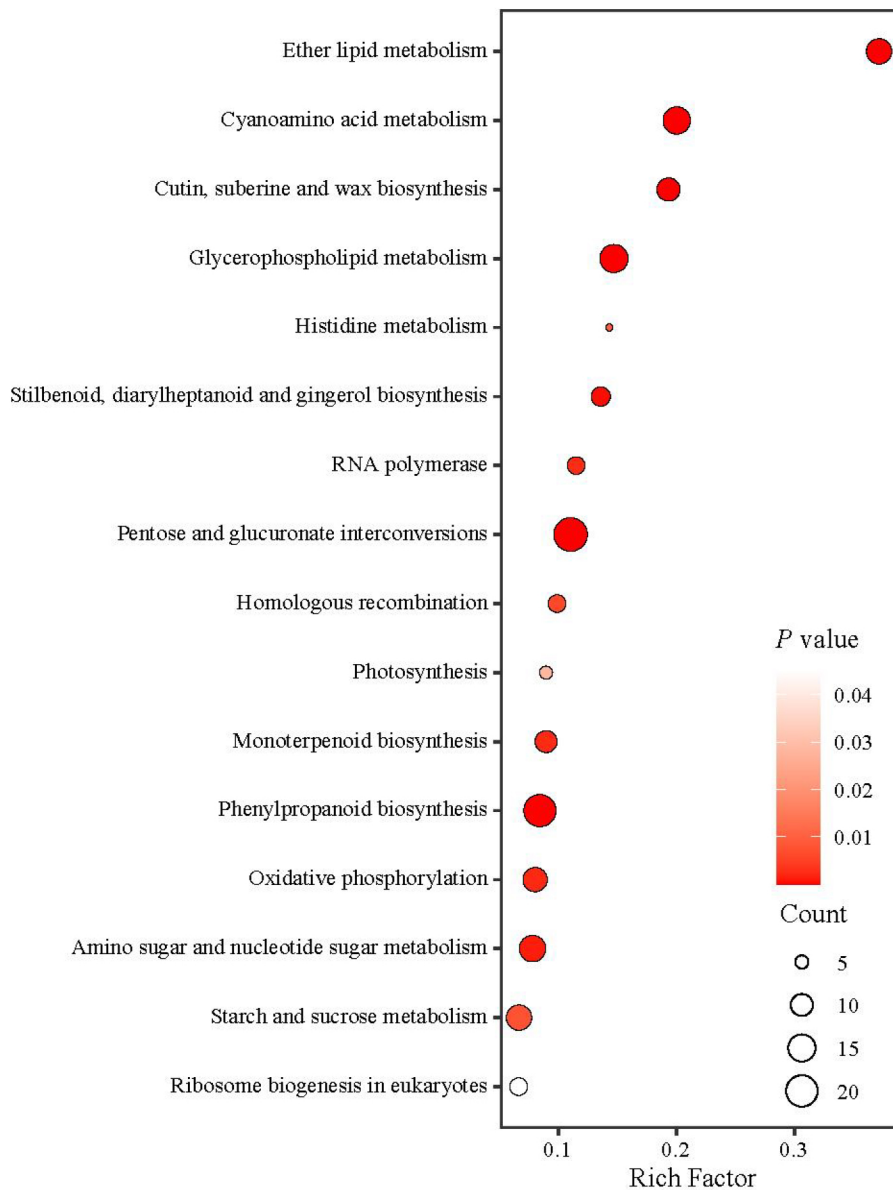

Supplement: Supplementary Figure 1 — The position of telomere on 12 chromosomes of Chinese chestnut. [file DataSheet1.zip › Figure S6.pdf]

## Type of duplications

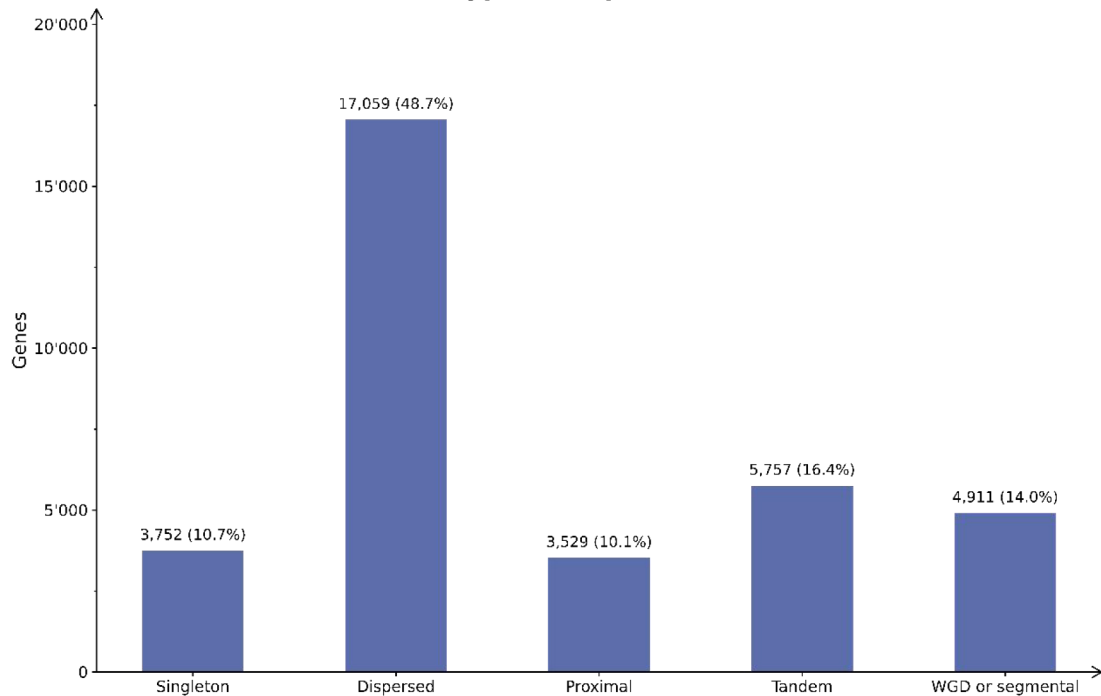

Supplement: Supplementary Figure 1 — The position of telomere on 12 chromosomes of Chinese chestnut. [file DataSheet1.zip › Figure S7.pdf]
